# Cardiovascular outcomes in patients with chronic kidney disease and COVID-19: a multi-regional data-linkage study

**DOI:** 10.1183/13993003.03168-2021

**Published:** 2022-11-10

**Authors:** Emilie J. Lambourg, Peter J. Gallacher, Robert W. Hunter, Moneeza Siddiqui, Eve Miller-Hodges, James D. Chalmers, Dan Pugh, Neeraj Dhaun, Samira Bell

**Affiliations:** 1 Division of Population Health and Genomics, University of Dundee, Dundee, UK; 2 BHF/University Centre for Cardiovascular Science, University of Edinburgh, Edinburgh, UK; 3 Dept of Renal Medicine, Royal Infirmary of Edinburgh, Edinburgh, UK; 4 Dept of Molecular and Clinical Medicine, University of Dundee, Dundee, UK; 5 These authors contributed equally; 6 These authors contributed equally

## Abstract

**Background:**

Data describing cardiovascular outcomes in patients with coronavirus disease 2019 (COVID-19) and chronic kidney disease (CKD) are lacking. We compared cardiovascular outcomes of patients with and without COVID-19, stratified by CKD status.

**Methods:**

This retrospective, multi-regional data-linkage study utilised individual patient-level data from two Scottish cohorts. All patients tested for severe acute respiratory syndrome coronavirus 2 (SARS-CoV-2) in Cohort 1 between 1 February 2020 and 31 March 2021 and in Cohort 2 between 28 February 2020 and 8 February 2021 were included.

**Results:**

Overall, 86 964 patients were tested for SARS-CoV-2. There were 36 904 patients (mean±sd age 61±21 years; 58.1% women; 15.9% CKD; 10.1% COVID-19 positive) in Cohort 1 and 50 060 patients (mean±sd age 63±20 years; 62.0% women; 16.4% CKD; 9.1% COVID-19 positive) in Cohort 2. In CKD patients, COVID-19 increased the risk of cardiovascular death by more than two-fold within 30 days (cause-specific hazard ratio (csHR) meta-estimate 2.34, 95% CI 1.83–2.99) and by 57% at the end of study follow-up (csHR meta-estimate 1.57, 95% CI 1.31–1.89). Similarly, the risk of all-cause death in COVID-19 positive *versus* negative CKD patients was greatest within 30 days (HR 4.53, 95% CI 3.97–5.16). Compared with patients without CKD, those with CKD had a higher risk of testing positive (11.5% *versus* 9.3%). Following a positive test, CKD patients had higher rates of cardiovascular death (11.1% *versus* 2.7%), cardiovascular complications and cardiovascular hospitalisations (7.1% *versus* 3.3%) than those without CKD.

**Conclusions:**

COVID-19 increases the risk of cardiovascular and all-cause death in CKD patients, especially in the short-term. CKD patients with COVID-19 are also at a disproportionate risk of cardiovascular complications than those without CKD.

## Abstract 2

**COVID-19 significantly increases the risk of cardiovascular complications and death in patients with CKD, especially in the short-term. COVID-19 vaccination and cardiovascular risk reduction strategies must be prioritised for all patients with CKD.** https://bit.ly/3MAY7Wg

## Introduction

Coronavirus disease 2019 (COVID-19), the disease caused by severe acute respiratory syndrome coronavirus 2 (SARS-CoV-2) [1], has had an unprecedented public health, societal and economic impact. Disease severity in patients with COVID-19 can vary markedly, from no symptoms or a mild respiratory illness to life-threatening pulmonary and extrapulmonary complications and death [2]. Several factors have been identified that increase disease severity. These include older age, male sex, social deprivation, obesity and comorbidities, such as cardiovascular disease and chronic kidney disease (CKD) [3–8]. The risk of critical illness and death following COVID-19 increases as kidney function declines, such that patients with the most advanced CKD have the poorest outcomes [4, 9–11].

The commonest complication of CKD is cardiovascular disease [12]. As estimated glomerular filtration rate (eGFR) declines, the risk of cardiovascular disease and major adverse cardiovascular events increases [13]. While pre-existing cardiovascular disease is a risk factor for COVID-19 severity, several studies have also suggested that myocardial injury and cardiovascular complications are common following COVID-19 and associated with worse outcomes for these patients [14–16]. However, data describing the nature and frequency of cardiovascular outcomes in patients with CKD and COVID-19 are lacking. Specifically, it is unclear how COVID-19 modifies the existing cardiovascular risk in patients with CKD in the short and medium term. In this unique multi-regional data-linkage study, we evaluated the clinical characteristics and cardiovascular outcomes of patients with and without COVID-19, stratified by the presence or absence of CKD.

An overview of the study methodology and main results is provided as a graphical abstract in the supplementary material.

## Methods

### Patient population and study design

We conducted a retrospective, multi-regional study utilising linked individual patient-level data from two cohorts in Scotland, UK. All SARS-CoV-2 PCR tests performed during the relevant time periods described for each cohort were included, regardless of whether they were from a hospital or community setting and irrespective of their indication (*e.g.* clinical, screening or research). Each patient's index COVID-19 status was defined as follows. 1) COVID-19 positive: where a patient first recorded a positive SARS-CoV-2 test result, they were included as an index positive episode occurring on that date. In this subpopulation, prior or subsequent negative SARS-CoV-2 test results within the study period were excluded, as were subsequent positive SARS-CoV-2 test results. 2) COVID-19 negative: where a patient first recorded a negative SARS-CoV-2 test result, but in the absence of any prior or subsequent positive test result, they were included as an index negative episode occurring on that date. In this subpopulation, subsequent negative SARS-CoV-2 test results were also excluded. The date of each index positive or negative episode was assigned as the index date.

### Study cohorts

#### Cohort 1

All patients who had a SARS-CoV-2 test in the NHS Lothian Health Board between 1 February 2020 and 31 March 2021 were identified. Positive and negative COVID-19 episodes were linked with regional electronic patient and biochemistry records and national hospitalisation, dispensed community prescription and death records within the DataLoch Repository and Safe Haven (University of Edinburgh/NHS Lothian) (supplementary material).

#### Cohort 2

All patients who had had a measure of serum creatinine in the NHS Fife or Tayside Health Boards since 1 April 2009, and subsequently had a SARS-CoV-2 test between 28 February 2020 and 8 February 2021, were identified. Positive and negative COVID-19 episodes were defined as for Cohort 1 before being linked with national hospitalisation, dispensed community prescription and death records within the Health Informatics Centre Safe Haven (University of Dundee/NHS Fife and Tayside) (supplementary material) [17].

### Determination of patient demographics, CKD status, comorbidities and causes of death

Patient age, sex and socioeconomic status were determined from linked hospitalisation records. Socioeconomic status was defined according to the Scottish Index of Multiple Deprivation, a validated measure of social deprivation determined by factors related to residential address (postcode) (supplementary material) [18]. CKD status was determined at the time of index SARS-CoV-2 test utilising the same, previously validated criteria for both study cohorts [19]. eGFRs were calculated for all serum creatinine results using the Chronic Kidney Disease Epidemiology (CKD-EPI) Collaboration equation [20]. CKD was defined when a patient's most recent eGFR was <60 mL·min^−1^·1.73 m^−2^ and at least one value obtained >90 days prior was also <60 mL·min^−1^·1.73 m^−2^. Using the eGFR value closest to the index date for each patient, CKD stage was classified according to Kidney Disease: Improving Global Outcomes (KDIGO) guidelines [21]. Patients with kidney failure requiring kidney replacement therapy (*i.e.* haemodialysis, peritoneal dialysis or kidney transplantation) were identified from record linkage with regional or national renal registries (Cohort 1: VitalData; Cohort 2: Scottish Renal Registry). Those patients with only a single measure of eGFR <60 mL·min^−1^·1.73 m^−2^ prior to their index test were excluded (supplementary figure S1).

Patient comorbidities (*i.e.* angina, atrial fibrillation, cancer, chronic liver disease, chronic lower respiratory disease, heart failure, myocardial infarction and stroke) were defined from International Classification of Diseases (ICD) codes associated with hospitalisations during a 5-year “lookback” period prior to the index SARS-CoV-2 test (supplementary material). Diabetes status was obtained *via* record linkage with a national diabetes registry (Scottish Care Information – Diabetes Collaboration) [22]. History of prescribed medications was determined from the community prescribing records of individual patients during the 6 months preceding their index SARS-CoV-2 test. Causes of death were determined following the identification of relevant ICD codes in linked National Records of Scotland death records (supplementary material).

### Study follow-up and outcomes

Patients were followed-up from the index date until either their date of death or 30 April 2021 (Cohort 1) or 20 May 2021 (Cohort 2), whichever came first. For both cohorts, information relating to primary and secondary causes of death was obtained *via* record linkage with the National Records of Scotland death registry (supplementary material). Primary outcomes included cardiovascular, COVID-19-related and all-cause death. Secondary outcomes included subsequent fatal and nonfatal myocardial infarction, heart failure or stroke, hospitalisation for cardiovascular diagnoses, hospitalisation for any reason, and length of hospital stay. For each cohort, outcomes were reported at 30 days, 90 days and to the end of study follow-up.

### Statistical analysis

#### Summary statistics

Baseline clinical characteristics and crude outcomes for all index SARS-CoV-2 tests included in each cohort were summarised according to CKD and COVID-19 status. Continuous variables were presented as median (interquartile range) and categorical variables were presented as percentage. Where appropriate, groupwise comparisons were performed using Chi-squared tests.

#### Covariate-balanced propensity scoring and regression modelling

With the aim of obtaining unconfounded estimates, we utilised a “doubly robust” estimator with bootstrapped standard errors and 95% confidence intervals in our evaluation of the primary outcomes (supplementary material) [23]. This approach combines a multivariable outcome regression model with weighting by the covariate-balanced propensity score (CBPS). For the primary analysis, Cox regression was used to explore the association between COVID-19 status (the primary exposure) and cardiovascular and all-cause death (the primary outcomes). For the secondary analysis, Cox regression was used to explore the association between CKD status (the primary exposure) and cardiovascular, COVID-19 and all-cause death (the primary outcomes). Confounders were specified *a priori*, and included age, sex, socioeconomic status, comorbidities (*i.e.* angina, myocardial infarction, heart failure, stroke, diabetes, cancer, chronic respiratory disease and chronic liver disease) and selected current medication (angiotensin converting enzyme (ACE) inhibitors/angiotensin receptor blockers (ARBs) and immunosuppressant therapy) based on their potential relevance to COVID-19 outcomes [24, 25]. For each primary outcome, hazard ratios derived from CBPS-weighted, adjusted multivariable models in individual cohorts were pooled using a fixed effects model to obtain an overall meta-estimate (supplementary material). Finally, a sensitivity analysis was also performed to evaluate the effect of restricting the cohorts to those patients not hospitalised either in the week before or in the 2 weeks following their index COVID-19 test. All data were analysed using R version 3.6.2 (www.r-project.org).

### Ethical approval

The study was performed with the approvals of local research ethics committees and delegated Caldicott Guardians for the NHS Fife, Lothian and Tayside Health Boards, in accordance with the Declaration of Helsinki (supplementary material). Data provision and linkage were carried out by the DataLoch (University of Edinburgh/NHS Lothian; www.dataloch.org) and University of Dundee Health Informatics Centre (www.dundee.ac.uk/hic) within ISO 27001 and Scottish Government accredited secure Safe Havens for Cohorts 1 and 2, respectively. Patient consent was not sought as the study utilised fully anonymised data. Our analysis code is publicly available *via* GitHub (https://github.com/pgallach/covid_renal.git).

## Results

### Study cohorts

In total, 86 964 out of 102 894 (84.5%) patients tested for SARS-CoV-2 were included in the study (supplementary figure S1). There were 36 904 patients (mean±sd age 61±21 years; 58.1% women) in Cohort 1 and 50 060 patients (mean±sd age 63±20 years; 62.0% women) in Cohort 2. Overall, the distribution of patient demographics and clinical characteristics between each cohort was similar. CKD was present in 15.9% (5853 out of 36 904) of patients in Cohort 1 and 16.4% (8201 out of 50 060) of patients in Cohort 2 (supplementary table S1), while a positive SARS-CoV-2 test was recorded in 10.1% (3731 out of 36 904) and 9.1% (4556 out of 50 060) of patients in Cohorts 1 and 2, respectively (supplementary table S2). In both cohorts, CKD was more common in patients with COVID-19 than in those without (Cohort 1: 19.7% *versus* 15.4%; Cohort 2: 19.0% *versus* 16.1%).

### Baseline characteristics of patients with CKD according to COVID-19 status

In patients with CKD, those with COVID-19 were older and more socially deprived than those without COVID-19 (table 1). Although SARS-CoV-2 testing was performed more frequently in women than in men with CKD (Cohort 1: 53.8% *versus* 46.2%; Cohort 2: 59.5% *versus* 40.5%) (supplementary table S3), the proportion of women testing positive and negative was similar (Cohort 1: 52.3% *versus* 54.0%; Cohort 2: 58.0% *versus* 59.7%) (table 1). Patients with CKD who tested positive had higher rates of cardiovascular comorbidity but similar eGFR compared with patients who tested negative. Despite this, across both cohorts, patients with CKD who tested positive were less likely to be prescribed an ACE inhibitor or ARB at the time of their index SARS-CoV-2 test than those who tested negative (Cohort 1: 35.8% *versus* 41.5%; Cohort 2: 25.2% *versus* 36.7%) (table 1). The baseline characteristics of patients without CKD summarised according to COVID-19 status (table 1), and of patients with and without COVID-19 summarised according to CKD status (supplementary table S4), are described in the supplementary material.

**TABLE 1:** Clinical characteristics of patients included in Cohorts 1 and 2, grouped according to chronic kidney disease (CKD)^#^ and COVID-19 status

	**Cohort 1**				**Cohort 2**			
	**CKD**		**No CKD**		**CKD**		**No CKD**	
	**COVID-19 positive**	**COVID-19 negative**	**COVID-19 positive**	**COVID-19 negative**	**COVID-19 positive**	**COVID-19 negative**	**COVID-19 positive**	**COVID-19 negative**
**Patients**	734	5119	2997	28 054	865	7336	3691	38 168
**Age (years)**	81±11	79±12	59±21	57±20	84±10	82±10	59±20	59±19
**Sex**								
Women	384 (52.3)	2764 (54.0)	1726 (57.6)	16 584 (59.1)	502 (58.0)	4377 (59.7)	2453 (66.5)	23 696 (62.1)
Men	350 (47.7)	2355 (46.0)	1271 (42.4)	11 470 (40.9)	363 (42.0)	2959 (40.3)	1238 (33.5)	14 472 (37.9)
**SIMD quintile**								
1 (most deprived)	103 (14.0)	616 (12.0)	476 (15.9)	4263 (15.2)	162 (18.7)	1160 (15.8)	831 (22.5)	7522 (19.7)
2	207 (28.2)	1280 (25.0)	795 (26.5)	6872 (24.5)	185 (21.4)	1391 (19.0)	747 (20.2)	7264 (19.0)
3	113 (15.4)	920 (18.0)	532 (17.8)	4945 (17.6)	182 (21.0)	1491 (20.3)	665 (18.0)	7187 (18.8)
4	112 (15.3)	889 (17.4)	519 (17.3)	4873 (17.4)	216 (25.0)	1977 (26.9)	905 (24.5)	9932 (26.0)
5 (least deprived)	198 (27.0)	1404 (27.4)	675 (22.0)	6883 (24.5)	120 (13.9)	1317 (18.0)	543 (14.7)	6262 (16.4)
**Coexisting medical conditions**								
Angina	91 (12.4)	571 (11.2)	125 (4.2)	1130 (4.0)	69 (8.0)	533 (7.3)	98 (2.7)	960 (2.5)
Atrial fibrillation^¶^					207 (23.9)	1444 (19.7)	215 (5.8)	2003 (5.2)
Myocardial infarction	124 (16.9)	807 (15.8)	178 (5.9)	1765 (6.3)	91 (10.5)	744 (10.1)	96 (2.6)	1239 (3.2)
Heart failure	196 (26.7)	1152 (22.5)	135 (4.5)	1268 (4.5)	133 (15.4)	847 (11.5)	84 (2.3)	740 (1.9)
Stroke	127 (17.3)	704 (13.8)	257 (8.6)	1729 (6.2)	123 (14.2)	671 (9.1)	237 (6.4)	1454 (3.8)
Diabetes	275 (37.5)	1819 (35.5)	491 (16.4)	3592 (12.8)	277 (32.0)	2388 (32.6)	589 (16.0)	5831 (15.3)
Cancer	159 (21.7)	1372 (26.8)	384 (12.8)	5373 (19.2)	98 (11.3)	858 (11.7)	154 (4.2)	2415 (6.3)
Chronic lower respiratory disease	223 (30.4)	1418 (27.7)	809 (27.0)	7411 (26.4)	232 (26.8)	1877 (25.6)	634 (17.2)	7797 (20.4)
Chronic liver disease	27 (3.7)	213 (4.2)	77 (2.6)	984 (3.5)	21 (2.4)	171 (2.3)	40 (1.1)	590 (1.5)
**Renal history**								
Kidney failure	35 (4.8)	302 (5.9)			29 (3.4)	211 (2.9)		
Baseline eGFR (mL·min^−1^·1.73 m^−2^)	40±14	42±14	90±19	91±18	43±12	44±12	93±18	94±19
Baseline eGFR category (mL·min^−1^·1.73 m^−2^)								
≥90			1396 (46.6)	14 090 (50.2)			2020 (54.7)	20 764 (54.4)
60–89			1601 (53.4)	13 964 (49.8)			1671 (45.3)	17 404 (45.6)
45–59	314 (42.8)	2394 (46.8)			436 (50.4)	3828 (52.2)		
30–44	236 (32.2)	1632 (31.9)			270 (31.2)	2279 (31.1)		
15–29	118 (16.1)	669 (13.1)			105 (12.1)	809 (11.0)		
<15	66 (9.0)	424 (8.3)			54 (6.2)	420 (5.7)		
**Current medication**								
ACE inhibitor or ARB	263 (35.8)	2126 (41.5)	674 (22.5)	5959 (21.2)	218 (25.2)	2695 (36.7)	589 (16.0)	7171 (18.8)
Aspirin^¶^					157 (18.1)	1599 (21.8)	299 (8.1)	3437 (9.0)
Other antiplatelet agent^¶^					128 (14.8)	861 (11.7)	247 (6.7)	2099 (5.5)
β-blockers^¶^					300 (34.7)	2655 (36.2)	497 (13.5)	5862 (15.4)
Immunosuppressants	27 (3.7)	201 (3.9)	66 (2.2)	566 (2.0)	11 (1.3)	151 (2.1)	13 (0.4)	246 (0.6)
Loop diuretic^¶^					303 (35.0)	2312 (31.5)	264 (7.1)	2553 (6.7)
MRA^¶^					66 (7.6)	558 (7.6)	64 (1.7)	644 (1.7)
Novel oral anticoagulant^¶^					142 (16.4)	1136 (15.5)	154 (4.2)	2018 (5.3)
Warfarin^¶^					43 (5.0)	499 (6.8)	45 (1.2)	651 (1.7)

Data are presented as n, mean±sd or n (%). SIMD: Scottish Index of Multiple Deprivation; eGFR: estimated glomerular filtration rate; ACE: angiotensin converting enzyme; ARB: angiotensin receptor blocker; MRA: mineralocorticoid receptor antagonist. ^#^: see Methods for definition of CKD status; ^¶^: data not available for Cohort 1.

### Outcomes of patients with CKD according to COVID-19 status

**FIGURE 1:**
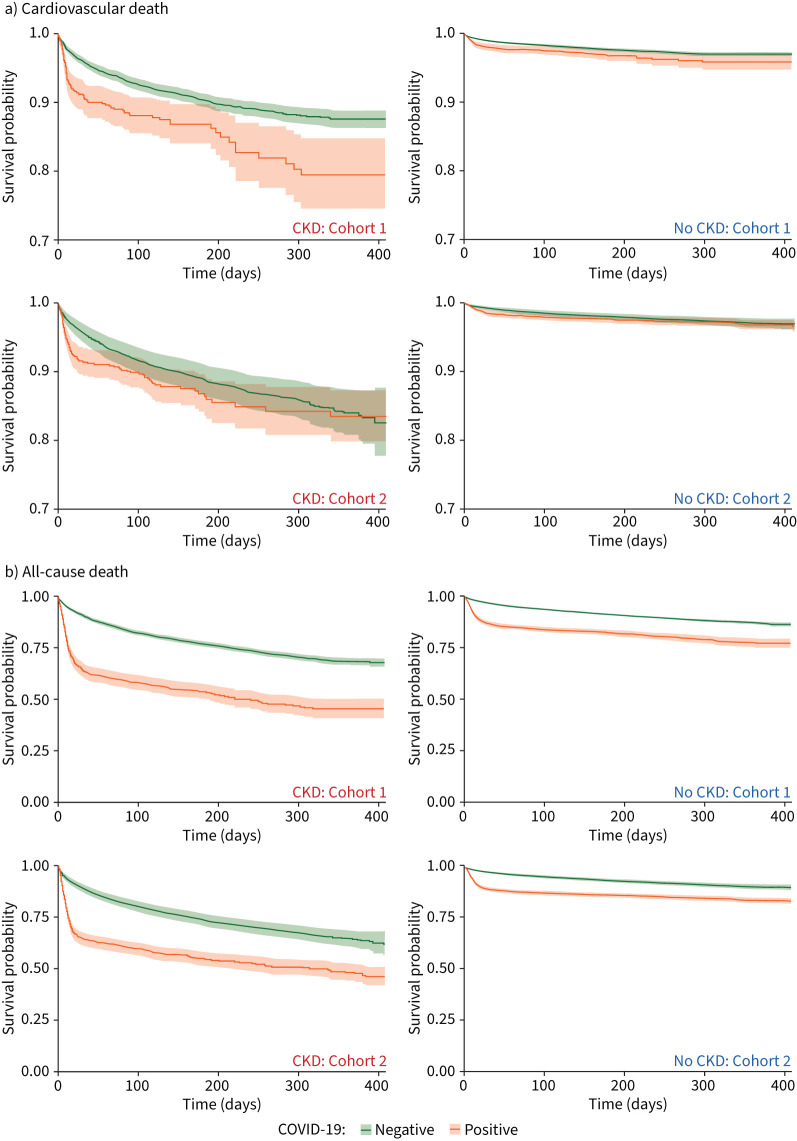
Survival curves for a) cardiovascular and b) all-caused death according to COVID-19 status (positive *versus* negative) for patients with chronic kidney disease (CKD) (left columns) and patients without CKD (right columns) in Cohorts 1 (top rows) and 2 (bottom rows) Note: *y*-axis scales are different for a) cardiovascular and b) all-cause death plots.

**FIGURE 2:**
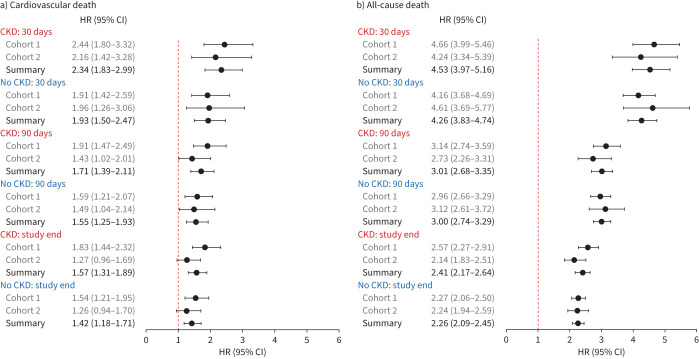
Forest plots summarising adjusted hazard ratios (HR) from Cohorts 1 and 2 and associated pooled meta-estimates for a) cardiovascular and b) all-cause death according to COVID-19 status (positive *versus* negative) for patients with chronic kidney disease (CKD) and patients without CKD at 30 days, 90 days and to the end of study follow-up.

In patients with CKD, the crude rate of cardiovascular death at 30 days in those with COVID-19 was double that of patients without COVID-19 (Cohort 1: 7.8% *versus* 3.4%; Cohort 2: 7.2% *versus* 3.5%) (table 2, figure 1a and supplementary table S5). After balancing differences in covariates between positive and negative patients (supplementary figure S2), and following adjustment for confounders, this increase in short-term cardiovascular risk persisted and was more than two-fold higher in positive than in negative patients at 30 days (cause-specific hazard ratio (csHR) meta-estimate 2.34, 95% CI 1.83–2.99) (figure 2a). By the end of study follow-up, the difference in cardiovascular risk between positive and negative patients with CKD had narrowed (csHR meta-estimate 1.57, 95% CI 1.31–1.89) (figures 1a and 2a).

**TABLE 2:** Outcomes of patients included in Cohorts 1 and 2, grouped according to chronic kidney disease (CKD) and COVID-19 status

	**Cohort 1**				**Cohort 2**			
	**CKD**		**No CKD**		**CKD**		**No CKD**	
	**COVID-19 positive**	**COVID-19 negative**	**COVID-19 positive**	**COVID-19 negative**	**COVID-19 positive**	**COVID-19 negative**	**COVID-19 positive**	**COVID-19 negative**
**Patients**	734	5119	2997	28 054	865	7336	3691	38 168
**Primary outcomes**								
Cardiovascular death								
30 days	57 (7.8)	172 (3.4)	57 (1.9)	254 (0.9)	62 (7.2)	254 (3.5)	55 (1.5)	316 (0.8)
90 days	72 (9.8)	290 (5.7)	68 (2.3)	377 (1.3)	72 (8.3)	453 (6.2)	70 (1.9)	515 (1.3)
End of study follow-up	86 (11.7)	426 (8.3)	86 (2.9)	542 (1.9)	92 (10.6)	730 (10.0)	94 (2.5)	840 (2.2)
COVID-19-related death								
30 days	250 (34.1)		377 (12.6)		295 (34.1)		397 (10.8)	
90 days	267 (36.4)		404 (13.5)		313 (36.2)		423 (11.5)	
End of study follow-up	270 (36.8)		407 (13.6)		318 (36.8)		427 (11.6)	
All-cause death								
30 days	260 (35.4)	442 (8.6)	399 (13.3)	974 (3.5)	302 (34.9)	642 (8.8)	405 (11.0)	1025 (2.7)
90 days	304 (41.4)	803 (15.7)	473 (15.8)	1702 (6.1)	342 (39.5)	1157 (15.8)	466 (12.6)	1782 (4.7)
End of study follow-up	346 (47.1)	1246 (24.3)	541 (18.1)	2688 (9.6)	397 (45.9)	1880 (25.6)	547 (14.8)	3007 (7.9)
**Secondary outcomes**								
Fatal/nonfatal myocardial infarction								
30 days	7 (1.0)	132 (2.6)	8 (0.3)	254 (0.9)	9 (1.0)	154 (2.1)	NA	386 (1.0)
90 days	8 (1.1)	155 (3.0)	9 (0.3)	293 (1.0)	9 (1.0)	193 (2.6)	9 (0.2)	428 (1.1)
End of study follow-up	11 (1.5)	190 (3.7)	15 (0.5)	351 (1.3)	13 (1.5)	248 (3.4)	11 (0.3)	505 (1.3)
Fatal myocardial infarction								
End of study follow-up	NA	62 (1.2)	NA	74 (0.3)	7 (0.8)	113 (1.5)	10 (0.3)	142 (0.4)
Fatal/nonfatal heart failure								
30 days	16 (2.2)	117 (2.3)	NA	133 (0.5)	21 (2.4)	348 (4.7)	6 (0.2)	362 (0.9)
90 days	21 (2.9)	168 (3.3)	8 (0.3)	166 (0.6)	23 (2.7)	423 (5.8)	8 (0.2)	414 (1.1)
End of study follow-up	24 (3.3)	226 (4.4)	10 (0.3)	212 (0.8)	28 (3.2)	496 (6.8)	14 (0.4)	478 (1.3)
Fatal heart failure								
End of study follow-up	18 (2.5)	88 (1.7)	NA	60 (0.2)	20 (2.3)	182 (2.5)	11 (0.3)	93 (0.2)
Fatal/nonfatal stroke								
30 days	10 (1.4)	96 (1.9)	18 (0.6)	348 (1.2)	15 (1.7)	249 (3.4)	31 (0.8)	574 (1.5)
90 days	13 (1.8)	121 (2.4)	23 (0.8)	415 (1.5)	19 (2.2)	294 (4.0)	38 (1.0)	648 (1.7)
End of study follow-up	16 (2.2)	158 (3.1)	28 (0.9)	487 (1.7)	25 (2.9)	348 (4.7)	44 (1.2)	744 (1.9)
Fatal stroke								
End of study follow-up	6 (0.8)	57 (1.1)	14 (0.5)	135 (0.5)	NA	263 (3.6)	NA	410 (1.1)
Fatal/nonfatal pulmonary embolism								
End of study follow-up	NA	60 (1.2)	15 (0.5)	286 (1.0)	7 (0.8)	87 (1.2)	17 (0.5)	370 (1.0)
Fatal pulmonary embolism								
End of study follow-up	NA	11 (0.2)	NA	20 (0.1)	NA	11 (0.1)	NA	41 (0.1)
Atrial fibrillation hospitalisations								
30 days	NA	189 (3.7)	7 (0.2)	461 (1.6)	7 (0.8)	259 (3.5)	10 (0.3)	626 (1.6)
90 days	12 (1.6)	225 (4.4)	13 (0.4)	557 (2.0)	12 (1.4)	303 (4.1)	13 (0.3)	714 (1.9)
End of study follow-up	15 (2.0)	292 (5.7)	18 (0.6)	676 (2.4)	24 (2.8)	435 (5.9)	24 (0.6)	939 (2.5)
Cardiovascular hospitalisations								
30 days	28 (3.8)	630 (12.3)	53 (1.8)	1847 (6.6)	41 (4.7)	1311 (17.9)	80 (2.2)	3076 (8.1)
90 days	41 (5.6)	765 (14.9)	82 (2.7)	2175 (7.8)	49 (5.7)	1483 (20.2)	100 (2.7)	3381 (8.9)
End of study follow-up	54 (7.4)	958 (18.7)	105 (3.5)	2549 (9.1)	60 (6.9)	1636 (22.3)	119 (3.2)	3685 (9.7)
All hospitalisations								
30 days	274 (37.3)	2928 (57.2)	896 (29.9)	13 361 (47.6)	359 (41.5)	5063 (69.0)	899 (24.4)	18 562 (48.6)
90 days	335 (45.6)	3408 (66.6)	1128 (37.6)	15 767 (56.2)	388 (44.9)	5267 (71.8)	970 (26.3)	19 242 (50.4)
End of study follow-up	386 (52.6)	3887 (75.9)	1311 (43.7)	17 952 (64.0)	411 (47.5)	5429 (74.0)	1043 (28.3)	19 911 (52.2)
Length of stay (days)	12 (5–24)	5 (2–12)	7 (3–18)	3 (1–7)	9 (4–17)	6 (2–16)	5 (2–15)	2 (0–7)

Data are presented as n, n (%) or median (interquartile range). NA: not available (potentially identifiable or count data <5, redacted in order to protect patient confidentiality).

In patients with CKD, the risk of all-cause death at 30 days in those with COVID-19 was substantially higher than in those without COVID-19 (Cohort 1: 35.4% *versus* 8.6%; Cohort 2: 34.9% *versus* 8.8%) (table 2, figure 1b and supplementary table S5). In the fully adjusted models, the risk of all-cause death in patients with CKD and COVID-19 was increased more than four-fold at 30 days (HR meta-estimate 4.53, 95% CI 3.97–5.16) and by more than two-fold overall (HR meta-estimate 2.41, 95% CI 2.17–2.64) compared with those with CKD testing negative. In contrast, cardiovascular complications and subsequent hospitalisations were lower in positive than in negative patients with CKD at 30 days, 90 days and to the end of study follow-up (table 2).

In a sensitivity analysis restricted to patients with CKD not hospitalised either in the week before or in the 2 weeks following their index COVID-19 test, overall rates of cardiovascular and all-cause death were lower than those reported in the primary analysis (supplementary table S6). However, COVID-19 was associated with a significantly increased risk of cardiovascular and all-cause death at all time-points, especially in the short-term; a pattern which was comparable to the primary analysis (supplementary table S7).

### Outcomes of patients with COVID-19 according to CKD status and eGFR

In patients with COVID-19, those with CKD had a higher risk of cardiovascular death than those without CKD (csHR meta-estimate 1.64, 95% CI 1.29–2.10) (supplementary table S8, and supplementary figures S3 and S4). Similarly, CKD was associated with a significantly increased risk of all-cause death in patients testing positive (csHR meta-estimate 1.25, 95% CI 1.12–1.41) (supplementary table S8, and supplementary figures S3 and S4). When eGFR was analysed as a continuous variable, the risk of both cardiovascular and all-cause death increased as kidney function declined (supplementary figure S5).

In patients with COVID-19, CKD was associated with an increased risk of COVID-19-related death (csHR meta-estimate 1.27, 95% CI 1.12–1.43) (supplementary table S8 and supplementary figure S6). Again, the risk of COVID-19-related death increased significantly as eGFR declined, even after adjustment for confounders (supplementary figure S7). Rates of cardiovascular complications and subsequent hospitalisations were also higher in COVID-19 positive patients with CKD than without (supplementary table S8).

The outcomes of patients without CKD summarised according to COVID-19 status (table 2, and figures 1a and 2a), and of patients without COVID-19 summarised according to CKD status and eGFR (supplementary table S8, and supplementary figures S3 and S4), are described in the supplementary material.

## Discussion

In this multi-regional data-linkage study, we utilised a robust statistical approach combining multivariable outcome regression with propensity score weighting to evaluate the outcomes of 86 964 patients with and without CKD tested for SARS-CoV-2. Overall, one in 10 patients had a positive SARS-CoV-2 test. In patients with CKD, those with COVID-19 had a higher risk of cardiovascular and all-cause death than those without COVID-19 throughout follow-up, but especially within 30 days of SARS-CoV-2 testing. During this early period, patients with CKD and COVID-19 had a more than two-fold increased risk of cardiovascular death, and a more than four-fold increased risk of all-cause death, than CKD patients testing negative. Compared with patients without CKD, those with CKD were also more likely to test positive. Following a positive test, CKD patients had higher rates of cardiovascular complications, including hospitalisations, and cardiovascular death than those without CKD. Moreover, the risks of cardiovascular, COVID-19-related and all-cause death increased as kidney function declined.

Our study has several strengths. First, its multi-regional design combined high-fidelity, high-quality Scottish linked healthcare data from patients undergoing community- and hospital-based SARS-CoV-2 testing in three large NHS Health Boards (which together provide care for around 1.7 million people), irrespective of age, sex, socioeconomic, kidney function or hospitalisation status. Thus, the influence of case selection bias on our patient cohorts was minimised. Moreover, the accuracy and completion rates of the data sources used in this study were recently reported as 96% [26] and 99% [27], respectively. Second, we utilised routinely collected biochemistry data and criteria previously validated in electronic health records [19] to determine baseline kidney function, reducing the potential for misclassification of CKD status. Third, our inclusion of control populations, *i.e.* COVID-19 negative patients and patients without CKD, alongside our use of a “doubly robust” estimator (concomitant multivariable outcome regression and weighting by the propensity score), limited the influence of confounding bias in our analyses [23, 28].

While the majority of patients with COVID-19 are considered to have increased cardiovascular risk [29], few studies have examined the nature or extent of this risk in patients with CKD [30]. This is important given the well-recognised association between cardiovascular disease and CKD [12, 13]. Here, in around 14 000 nonhospitalised and hospitalised patients with CKD tested for SARS-CoV-2, we found that COVID-19 more than doubled the risk of cardiovascular death within 30 days and by 57% overall. Our secondary analysis showed that patients with COVID-19 and CKD had an increased risk of fatal and nonfatal myocardial infarction, heart failure and stroke compared with patients with COVID-19 but no CKD. In an adjusted model, CKD was also associated with a 64% increased risk of cardiovascular death in patients with COVID-19. In contrast, Rao *et al.* [31] investigated the risk of cardiovascular complications in patients with COVID-19 but found no increase in risk in patients with CKD compared with those without CKD. However, this study excluded nonhospitalised patients and patients who had tested negative for SARS-CoV-2, and relied on manual case note review to determine CKD status.

Our data add to the literature on COVID-19 outcomes in high-risk populations. However, a novel aspect of our approach is the inclusion of patients with CKD who tested negative for SARS-CoV-2. Of the few studies that have included such patients, all have identified COVID-19 as being strongly associated with a poor prognosis. Indeed, a recent meta-analysis found that COVID-19 increased the odds of death approximately six-fold in patients with CKD [32]. This is more in-line with the risk of all-cause death we report in patients with CKD and COVID-19 at 30 days post-index test, consistent with the fact that most studies included in this meta-analysis reported in-hospital mortality only. Thus, our study is unique in reporting both short- and medium-term patient outcomes. Our study is also less biased towards severe COVID-19 as we included both nonhospitalised and hospitalised patients, making our results more accurate, representative and informative for patients with all severities of CKD and COVID-19.

We found that, compared with patients without CKD, those with CKD were more likely to test positive for COVID-19. Thereafter, patients with COVID-19 and CKD had higher rates of fatal and nonfatal myocardial infarction, heart failure and stroke, cardiovascular hospitalisations, and cardiovascular, COVID-19-related, and all-cause death than patients with COVID-19 but no CKD. Few studies have reported on all these aspects. Our data are consistent with reports of an increasing incidence of COVID-19 as kidney function declines [33, 34]. A number of factors likely contribute to this increased risk of COVID-19 in CKD, including case ascertainment bias (*i.e.* patients with CKD are more likely to be tested for SARS-CoV-2), greater viral exposure secondary to more frequent healthcare encounters (*e.g.* in-centre haemodialysis) [11] and an underlying increased predisposition to infection due to altered immune response [35].

We recognise some limitations. First, our inability to account for selected variables (*e.g.* body mass index, smoking status and type of atrial fibrillation (*i.e.* paroxysmal *versus* permanent; nonvalvular or valvular)) means that we cannot exclude the potential for residual confounding. In addition, selected data relating to coexisting atrial fibrillation and prescribed cardiovascular medications were not available for patients included in Cohort 1. Second, rates of nonfatal cardiovascular complications, including atrial fibrillation and cardiovascular hospitalisations, may have been under-reported due to the competing risk of death in patients with COVID-19. Given the crude rates of nonfatal cardiovascular complications were generally higher in patients without COVID-19, this suggests that this might be the case. We overcame this when evaluating cardiovascular and COVID-19-related death by calculating cause-specific hazard ratios from our regression models. Third, we excluded patients with no record of kidney function and those with only a single eGFR <60 mL·min^−1^·1.73 m^−2^ during the biochemistry “lookback” period. Consequently, young or less comorbid patients (who are less likely to have had their kidney function tested) may be relatively under-represented. Finally, those patients with CKD who tested negative for SARS-CoV-2 were a relatively “sick” control group; their all-cause mortality was substantially higher than might be expected for the CKD population in general [36]. One explanation for this is that SARS-CoV-2 PCR testing was largely restricted to the in-hospital setting for much of the early phase of the COVID-19 pandemic in the UK [37]. To address this issue, and to illustrate the effect of including a more widely representative control group, we performed a sensitivity analysis restricted to those patients not hospitalised either in the week before or in the 2 weeks following their index COVID-19 test, and demonstrated a similar pattern of increased risk of cardiovascular and all-cause death in patients with COVID-19 compared with those without.

### Conclusions

Our unique and comprehensive analysis suggests that COVID-19 significantly increases the risk of cardiovascular complications and death in patients with CKD, especially in the short-term. There is an urgent need to prioritise COVID-19 vaccination and cardiovascular risk reduction strategies in all patients with CKD.

## Supplementary material

10.1183/13993003.03168-2021.Supp1 **Please note:** supplementary material is not edited by the Editorial Office, and is uploaded as it has been supplied by the author.
Appendix and supplementary material ERJ-03168-2021.Supplement
Visual abstract ERJ-03168-2021.Abstract

## Shareable PDF

10.1183/13993003.03168-2021.Shareable1 This one-page PDF can be shared freely online.
Shareable PDF ERJ-03168-2021.Shareable
