# Supplementary material for: Cardiovascular outcomes in patients with chronic kidney disease and COVID-19: a multi-regional data-linkage study
Source: Eur Respir J. 2022 Nov 10;60(5):2103168. doi: 10.1183/13993003.03168-2021 (PMC9101552; doi:10.1183/13993003.03168-2021)
Supplement: Supplementary file 1 [file ERJ-03168-2021.Supplement.pdf]

APPENDIX

Cardiovascular outcomes in patients with chronic kidney disease and COVID-19: a multi-regional data-linkage study

Emilie J. Lambourg*, PharmD,[1] Peter J. Gallacher*, MBChB MPH,[2] Robert W. Hunter, MBChB PhD,[2,3] Moneeza Siddiqui, PhD,[1] James Chalmers, MBChB PhD,[4] Dan Pugh, MBChB,[3] Neeraj Dhaun†, MBChB PhD,[2,3] Samira Bell†, MBChB MD[1,]

[1] Division of Population Health and Genomics, University of Dundee, Dundee, UK

[2] BHF/University Centre for Cardiovascular Science, University of Edinburgh, Edinburgh, UK

[3] Department of Renal Medicine, Royal Infirmary of Edinburgh, Edinburgh, UK

[4] Department of Molecular and Clinical Medicine, University of Dundee, Dundee, UK

[5] Scottish Renal Registry, Scottish Health Audits, Public Health & Intelligence, Glasgow, UK

*These authors contributed equally

†These authors contributed equally

Non-standard abbreviations

CHI – Community Health Index (number)

ICD – International Classification of Diseases

NRS – National Records for Scotland

SIMD – Scottish Index of Multiple Deprivation

SMR01 – Scottish Morbidity Record 01

Supplementary Methods

Study cohorts

Cohort 1

All patients who had a SARS-CoV-2 test in the NHS Lothian Health Board between February 1, 2020, and March 31, 2021 were identified. Positive and negative COVID-19 episodes were linked with regional electronic patient and biochemistry records (TrakCare software application, InterSystems Corporation, Cambridge, MA, USA) and national hospitalisation (Scottish Morbidity Record 01, Information Services Division, Scotland), dispensed community prescription (Prescribing Information System, Information Services Division, Scotland) and death records (National Records of Scotland, Scotland) within the DataLoch Repository and Safe Haven (University of Edinburgh/NHS Lothian, Edinburgh, Scotland).

Cohort 2

All patients who had had a measure of serum creatinine in the NHS Fife or Tayside Health Boards since April 1, 2009, and subsequently had a SARS-CoV-2 test between February 28, 2020, and February 8, 2021, were identified. Positive and negative COVID-19 episodes were defined as for Cohort 1 before being linked with national hospitalisation (Scottish Morbidity Record 01, Information Services Division, Scotland), dispensed community prescription (Prescribing Information System, Information Services Division, Scotland), and death records (National Records of Scotland, Scotland) within the Health Informatics Centre Safe Haven (University of Dundee/NHS Fife and Tayside, Dundee, Scotland).[17]

Data sources

Scottish Morbidity Record 01 (SMR01)

The Scottish Morbidity Record 01 (SMR01) general / acute inpatient and day case is an episode-based hospitalisation record, indexed by community health index (CHI) number, capturing all hospitalisations from non-obstetric and non-psychiatric specialties from all residents in Scotland. SMR01 are routinely collected healthcare data of high-quality in terms of consistency and national coverage. To ensure its quality, a set of validation rules is applied to all SMR01 data before release.(1) Across all SMR01

records, estimated completion and accuracy rates are 99% and 89%, respectively. For SMR01 records relating to cardiovascular diagnoses, the accuracy rate is 94.2%.(2)

Information contained in SMR01 data includes: date of admission and discharge date, admission type and where the episode took place, patient's conditions (ICD-10 coded) and interventions received (OPCS-4 coded). Up to 6 diagnoses are available to describe patients' conditions (main and five additional reasons for admission). All 6 positions were searched for presence of at least one relevant ICD-10 code over a 5-year look-back period prior to the index date to assess patient's baseline comorbidities. However, only the first 2 positions were searched to characterise cardiovascular hospitalisations occurring on or after the index date. Previously conducted sensitivity analyses validated the accuracy of restricting diagnosis coding to the first 2 out of 6 positions to ensure an optimal balance of diagnostic coding specificity and sensitivity.(3) Further information regarding SMR01 data is available on the website of the Information Services Division (National Services Scotland, NHS Scotland): https://www.ndc.scot.nhs.uk/National-Datasets/data.asp?SubID=5

National Records of Scotland (NRS) Death Records

The NRS is a CHI-indexed dataset covering all deaths in Scotland. Information on the death status, date of death, and causes of death were extracted and linked to the other records of patients tested for SARS-CoV-2 *via* their unique CHI number. Up to eleven different causes of death (the underlying cause and up to 10 additional causes) based on the amended cause of death text are recorded in the NRS data and coded using the ICD-10 classification. Besides the underlying cause, the additional causes of death are factors, diseases or injury that have contributed in some way to the occurrence of the death.(4) All positions were searched for relevant ICD-codes to identify COVID-19 related deaths whilst only the first two positions were considered to assess cardiovascular mortality. More information on the NRS data is

available on the website of the Information Services Division (National Services Scotland, NHS Scotland): https://www.ndc.scot.nhs.uk/National-Datasets/data.asp?SubID=13

The Scottish Renal Registry (SRR)

For the Tayside/Fife cohort, patients on chronic renal replacement therapy (RRT) were identified from the SRR. The SRR was established in 1991 with data backfilled to 1960 from European Renal Association-European Dialysis and Transplant Association (ERA-EDTA). It is a national registry of all patients receiving RRT (haemodialysis, peritoneal dialysis or kidney transplant) for end-stage kidney disease in Scotland, indexed by CHI number. The SRR has 100% unit and patient coverage. Data held by the registry include patient demographics, full RRT history, primary renal diagnosis (using ERA-EDTA codes) and monthly linkage with National Health Service Blood and Transplant (NHS BT) for transplant status. More information is available on the SRR website: https://www.srr.scot.nhs.uk/

The Scottish Care Information – Diabetes (SCI-D) database

The SCI-D is a CHI-indexed high-quality integrated database containing clinical information on over 99% of people with diagnosed diabetes in Scotland.(5) It is automatically updated from primary and secondary care systems, with almost all practices in Scotland participating to data collection. The dataset was used here as a population-based diabetes register since only the diagnosis date was extracted to identify all patients with a diagnosis of diabetes made prior to their index date.

Community prescribing

Medications prescribed over the 6-month period prior to the index date were used to determine presence of a baseline exposure to ACE-inhibitor/ARB, immunosuppressants or presence of a chronic respiratory disease in patients included in the cohort. The identification of a single relevant BNF code (see **Supplementary Text 1**) was necessary and sufficient to identify the comorbidity or drug exposure.

Determination of patient demographics, CKD status, comorbidities, causes of death and history of prescribed medications

Patient demographics

For both study cohorts, patient age, sex and social deprivation status were determined from linked hospitalisation records. Social deprivation status was defined according to the Scottish Index of Multiple Deprivation (SIMD) – a validated measure of social deprivation determined by factors related to residential address (zip code) (**Appendix**).(6) Patients were assigned an individual SIMD rank at the time of their index SARS-CoV-2 test. Based on these ranks, patients were assigned a SIMD quintile, where the first and fifth quintiles were considered the most and least deprived, respectively.(7)

Chronic kidney disease

Both study cohorts utilised the same criteria – validated in electronic health records and based on the timing and nature of prior serum creatinine results – to determine CKD status at the time of index SARS-CoV-2 test.(8) For patients included in Cohort 1, two years of prior serum creatinine data were used to inform CKD status using these criteria. For patients included in Cohort 2, all historic serum creatinine results dating back to April 1, 2009, were available for analysis.

In brief, eGFRs were calculated for all assay-corrected serum creatinine results using the Chronic Kidney Disease Epidemiology (CKD-EPI) Collaboration equation.(9) CKD was defined when a patient's most recent eGFR was <60 ml/min/1.73m^2 and at least one value obtained >90 days prior was also calculated as <60 ml/min/1.73m^2. Using the eGFR value closest to the index date for each patient, CKD stage was classified according to Kidney Disease: Improving Global Outcomes (KDIGO) guidelines.(10) Patients with kidney failure requiring kidney replacement therapy (i.e. haemodialysis, peritoneal dialysis or kidney transplantation) were identified from record linkage with regional or national renal registries (Cohort 1 – VitalData; Cohort 2 – Scottish Renal Registry). Those patients with only a single measure of eGFR <60 ml/min/1.73m^2 prior to their index test were excluded from further analysis (**Supplementary Figure 1**).

Comorbidities and causes of death

Patient comorbidities were defined from International Classification of Diseases (ICD) codes associated with hospitalisations during a 5-year 'lookback' period prior to the index SARS-CoV-2 test. For every

index COVID-19 episode identified in Cohorts 1 and 2, the following comorbidity data were extracted from linked hospitalisation records: (history of) angina, cancer, chronic liver disease, chronic lower respiratory disease, heart failure, myocardial infarction, and stroke. For Cohort 2, data relating to co-existing atrial fibrillation were also available. In addition, diabetes status was obtained *via* record linkage with a national diabetes registry (Scottish Care Information – Diabetes Collaboration).(11) Causes of death were determined following the identification of relevant ICD codes in linked National Records of Scotland death records.

History of prescribed medications

Anatomical Therapeutic Chemical (ATC) and British National Formulary (BNF) codes were used to identify dispensed prescriptions of angiotensin converting enzyme inhibitors or angiotensin receptor blockers (ACE-inhibitors/ARBs) and immunosuppressants in the community prescribing records of individual patients during the 6 months preceding their index SARS-CoV-2 test. For patients included in Cohort 2, prescription data relating to common cardiovascular medications (i.e. ACE-inhibitor, angiotensin receptor blockers, aspirin, antiplatelet agents, beta-blockers, loop diuretics, mineralocorticoid receptor antagonists, novel oral anticoagulants and warfarin) dispensed during the 6 months preceding each index SARS-CoV-2 test were also available.

Statistical analysis

Covariate-balanced propensity scoring and regression modelling

For our primary analysis, we sought to estimate the causal effect of COVID-19 on outcomes in patients with and without CKD, whilst for our secondary analysis, we sought to estimate the causal effect of CKD on outcomes in patients with and without COVID-19. With the aim of obtaining an unconfounded estimate, we utilised a 'doubly-robust' estimator with bootstrapped standard errors and 95% confidence intervals for the primary outcome analyses (**Appendix**).[(12)] This approach combines a multivariable outcome regression model with weighting by the covariate-balanced propensity score (CBPS). A key

strength of this method is that an unbiased effect estimate can still be obtained, even if one of the component models (either the outcome regression or CBPS model) has been mis-specified.[12, 13]

For the primary analysis, Cox regression was used to explore the association between COVID-19 status (the primary exposure) and cardiovascular and all-cause death (the primary outcomes). For the secondary analysis, Cox regression was used to explore the association between CKD status (the primary exposure) and cardiovascular, COVID-19 and all-cause death (the primary outcomes). For cardiovascular and COVID-19-related death, cause-specific hazard ratios were calculated to account for the competing risks of death from non-cardiovascular causes and death from non-COVID-19 causes, respectively.

For each analysis, we initially ran an unadjusted model before combining CBPS weighting and adjustment for several known confounders in a fully adjusted model. Using CBPS, covariates were balanced between exposure groups (COVID-19 and CKD status) in patients with and without CKD, and in patients with and without COVID-19 for the primary and secondary analyses, respectively.[14] Confounders were specified *a priori* and included age, sex, social demographic status, comorbidities (history of angina, myocardial infarction, heart failure, stroke, diabetes, cancer, chronic respiratory disease, chronic liver disease) and selected current medication (ACE-inhibitors/ARBs, immunosuppressant therapy) based on their potential relevance to COVID-19 outcomes.[15, 16]

For each primary outcome, hazard ratios derived from CBPS-weighted, fully-adjusted multivariable models in individual cohorts were pooled to obtain an overall meta-estimate. Both cohorts utilised similar patient populations based in the same country, whilst the design, definitions and overall methodology employed in each study cohort were also similar. Consequently, meta-estimates were computed using a fixed-effects model.

Thereafter, Cox regression models adjusting for the same confounders as before but with no CBPS-weighting were used to estimate the association between eGFR (primary exposure) and the risk of

cardiovascular, all-cause, and COVID-19-related death in patients with and without COVID-19. For this analysis, eGFR was modelled as a continuous variable with restricted cubic splines using knots placed at the 5th, 25th, 50th and 75th centiles of eGFR measures in each cohort, as described previously.[(17)]

Unadjusted and CBPS-weighted, multivariable Cox models were generated and fitted using the *cobalt*, *rms*, *survival*, and *WeightIt* packages, whilst meta-estimates were obtained using the *meta* package. All data were analysed using the R statistical programming language (Version 3.6.2, Vienna, Austria).

Missing data

Age, sex, comorbidity, prescribing, and outcome data were complete for all patients in both study cohorts. In Cohort 1, data relating to kidney function and social deprivation status were missing in 13.2% and 0.7% of all patient records, respectively. These were excluded from further analysis. In Cohort 2, data relating to social deprivation status were missing in 5.1%. For these records, hot deck imputation was implemented, with missing SIMD quintiles replaced by the observed value of another individual randomly chosen from all patients sharing a similar zip code.

Ethical (IRB) approval and statement of data transparency

The study was performed with the approvals of local Research Ethics Committees and delegated Caldicott Guardians for the NHS Fife, Lothian, and Tayside Health Boards, in accordance with the Declaration of Helsinki. All study methods and results were described and reported according to STROBE guidelines.(18) Data provision and linkage were carried out by the DataLoch (University of Edinburgh/NHS Lothian, https://www.dataloch.org/) and University of Dundee Health Informatics Centre (HIC, https://www.dundee.ac.uk/hic) within ISO27001 and Scottish Government accredited secure Safe Havens for Cohorts 1 and 2, respectively. HIC Standard Operating Procedures have been reviewed and approved by the NHS East of Scotland Research Ethics Service and consent was obtained from the NHS Fife Caldicott Guardian. Patient consent was not sought as the study utilised fully-anonymised data. To minimise the risk of disclosure, only summary data were extracted from each Safe Haven.

Individual-level data are available *via* application to the DataLoch and Health Informatics Centre. Our analysis code is publicly available here.

Supplementary Results

Baseline characteristics of patients without CKD according to COVID-19 status

In patients without CKD, differences in baseline characteristics according to COVID-19 status were less marked across both cohorts (**Table 1**). In patients who tested positive and negative, the proportion of women, rates of cardiovascular comorbidity and drug prescriptions, and eGFR were all similar.

Baseline characteristics of patients with COVID-19 according to CKD status

A positive SARS-CoV-2 test was more likely in patients with CKD than in those without CKD (Cohort 1: 12.5% *versus* 9.7%; Cohort 2: 10.5% *versus* 8.8%). In patients with COVID-19, those with CKD were older and had a greater burden of cardiovascular comorbidity than patients without CKD (**Supplementary Table 4**). The differences between patients with and without CKD were as marked in those without COVID-19.

Outcomes of patients without CKD according to COVID-19 status

In patients without CKD, the adjusted risk of cardiovascular death at 30 days was increased two-fold in patients with COVID-19 compared to patients testing negative (csHR meta-estimate 1.94, 95% CI 1.52 to 2.47) (**Table 2; Figures 1a & 2a**). By the end of study follow-up, the adjusted risk of cardiovascular death in positive *versus* negative patients without CKD had reduced (csHR meta-estimate 1.42, 95% CI 1.19 to 1.70). A similar pattern was evident for the risk of all-cause death in these patients (**Table 2; Figures 1b & 2b**).

Outcomes of patients without COVID-19 according to CKD status and eGFR

Across both cohorts, significant differences in outcomes between those with and without CKD were also evident in patients without COVID-19. CKD was associated with an increased risk of cardiovascular death (csHR meta-estimate 1.46, 95% CI 1.33 to 1.60) and all-cause death (csHR meta-estimate 1.24, 95% CI 1.18 to 1.31) (**Supplementary Table 6; Supplementary Figures 3 & 4**). Finally, CKD was also

associated with higher rates of cardiovascular complications and subsequent hospitalisations in these patients.

Supplementary Text 1. List of International Classification of Diseases and BNF codes employed in study.

Condition	ICD-10 Code
**Cancer**	
ICD-10	Any "C" code
**Chronic liver disease**	
ICD-10	B18, K70, K74.3, K74.4, K74.5, K74.6, I85.0, I85.9, I98.2, I98.3, K76, R18, Z94.4, K72.1, K71.3, K71.4, K71.5, K71.7
**Chronic respiratory disease***	
ICD-10	I27.8, I27.9 J40-45, J46, J47, J60-67, J68.4, J70.1, J70.3
BNF	030101, 030102, 030104, 0302, 030302
**COVID-19**	
ICD-10	U071, U072
**Cardiovascular diagnoses**	**ICD-10 Code**
**Angina**	
ICD-10	I201, I208, I209
**Heart failure**	
ICD-10	I11.0, I13.0, I13.2, I50
**Myocardial infarction**	
ICD-10	I21, I22, I23, I241, I252
**Stroke**	
ICD-10	G45, G46, I60-69
**Pulmonary embolism**	
ICD-10	I26.0, I26.9
**Community prescription data**	**ATC Code**
**Exposure to ACE-inhibitor/ARB**	
ATC	C09AA, C09CA
**Exposure to immunosuppressants**	
ATC	L04

Abbreviations: ACE-inhibitor – angiotensin converting enzyme inhibitor; ARB – angiotensin receptor blocker; ATC – anatomical therapeutic classification, BNF – British National Formulary, ICD – international classification of diseases. *For chronic respiratory disease, the presence of a single ICD-10 code in SMR01 data **OR** a single BNF code in community prescription data was necessary and sufficient to assess the presence of the comorbidity.

Supplementary Tables

Supplementary Table 1. Clinical characteristics of patients included in Cohorts 1 and 2.

	**COHORT 1**	**COHORT 2**
**Number of patients, n**	**36,904**	**50,060**
**Age, years**	61 (21)	63 (20)
**Sex**		
Women	21,458 (58.1)	31,028 (62.0)
Men	15,446 (41.9)	19,032 (38.0)
**SIMD quintile**		
1 (most deprived)	5,458 (14.8)	9,675 (19.3)
2	9,154 (24.8)	9,587 (19.2)
3	6,510 (17.6)	9,525 (19.0)
4	6,393 (17.3)	13,030 (26.0)
5 (least deprived)	9,145 (24.8)	8,242 (16.5)
**Co-existing medical conditions**		
Angina	1,917 (5.2)	1,660 (3.3)
Atrial fibrillation*	-	3,869 (7.7)
Myocardial infarction	2,874 (7.8)	2,170 (4.3)
Heart failure	2,751 (7.5)	1,804 (3.6)
Stroke	2,817 (7.6)	2,485 (5.0)
Diabetes	6,177 (16.7)	9,085 (18.1)
Cancer	7,288 (19.7)	3,525 (7.0)
Chronic lower respiratory disease	9,861 (26.7)	10,540 (21.1)
Chronic liver disease	1,301 (3.5)	822 (1.6)
**Renal history**		
CKD	5,853 (15.9)	8,201 (16.4)
End-stage kidney disease	337 (0.9)	240 (0.5)
Baseline eGFR	83 (25)	86 (26)
Baseline eGFR category		
≥90	15,486 (42.0)	22,784 (45.5)
60 – 89	15,565 (42.2)	19,075 (38.1)
45 – 59	2,708 (7.3)	4,264 (8.5)
30 – 44	1,868 (5.1)	2,549 (5.1)
15 – 29	787 (2.1)	914 (1.8)
≤15	490 (1.3)	474 (0.9)
**Current medication**		
ACE-inhibitor or ARB	9,022 (24.4)	10,673 (21.3)
Aspirin*	-	5,492 (11.0)
Other antiplatelet agent*	-	3,335 (6.7)
Beta-blockers*	-	9,314 (18.6)
Immunosuppressants	860 (2.3)	421 (0.8)
Loop diuretic*	-	5,432 (10.9)
Mineralocorticoid receptor antagonist*	-	1,332 (2.7)
Novel oral anticoagulant*	-	3,450 (6.9)
Warfarin*	-	1,238 (2.5)

Values are mean ± SD, n (%), or median (interquartile range). Abbreviations: ACE-inhibitor – angiotensin converting enzyme inhibitor; ARB – angiotensin receptor blocker; CKD – chronic kidney disease; eGFR – estimated glomerular filtration rate (ml/min/1.73 m^2); SIMD – Scottish index of multiple deprivation. *data not available for Cohort 1.

Supplementary Table 2. Clinical characteristics of patients included in Cohorts 1 and 2, grouped by COVID-19 status.

	**COHORT 1**		**COHORT 2**	
	**COVID-19 positive**	**COVID-19 negative**	**COVID-19 positive**	**COVID-19 negative**
**Number of patients, n**	**3,731**	**33,173**	**4,556**	**45,504**
**Age, years**	63 (22)	61 (20)	64 (21)	63 (20)
**Sex**				
Women	2,110 (56.6)	19,348 (58.3)	2,955 (64.9)	28,073 (61.7)
Men	1,621 (43.4)	13,825 (41.7)	1,601 (35.1)	17,431 (38.3)
**SIMD quintile**				
1 (most deprived)	579 (15.5)	4,879 (14.7)	993 (21.8)	8,682 (19.1)
2	1,002 (26.9)	8,152 (24.6)	932 (20.5)	8,655 (19.0)
3	645 (17.3)	5,865 (17.7)	847 (18.6)	8,678 (19.1)
4	631 (16.9)	5,762 (17.4)	1,121 (24.6)	11,909 (26.2)
5 (least deprived)	858 (23.0)	8,287 (25.0)	663 (14.6)	7,579 (16.7)
**Co-existing medical conditions**				
Angina	216 (5.8)	1,701 (5.1)	167 (3.7)	1,493 (3.3)
Atrial fibrillation*	-	-	422 (9.3)	3,447 (7.6)
Myocardial infarction	302 (8.1)	2,572 (7.8)	187 (4.1)	1,983 (4.4)
Heart failure	331 (8.9)	2,420 (7.3)	217 (4.8)	1,587 (3.5)
Stroke	384 (10.3)	2,433 (7.3)	360 (7.9)	2,125 (4.7)
Diabetes	766 (20.5)	5,411 (16.3)	866 (19.0)	8,219 (18.1)
Cancer	543 (14.6)	6,745 (20.3)	252 (5.5)	3,273 (7.2)
Chronic lower respiratory disease	1,032 (27.7)	8,829 (26.6)	866 (19.0)	9,674 (21.3)
Chronic liver disease	104 (2.8)	1,197 (3.6)	61 (1.3)	761 (1.7)
**Renal history**				
CKD	734 (19.7)	5,119 (15.4)	865 (19.0)	7,336 (16.1)
End-stage kidney disease	35 (0.9)	302 (0.8)	29 (0.6)	211 (0.5)
Baseline eGFR	80 (27)	84 (25)	84 (26)	86 (26)
Baseline eGFR category				
≥90	1,396 (37.4)	14,090 (42.5)	2,020 (44.3)	20,764 (45.6)
60 – 89	1,601 (42.9)	13,964 (42.1)	1,671 (36.7)	17,404 (38.2)
45 – 59	314 (8.4)	2,394 (7.2)	436 (9.6)	3,828 (8.4)
30 – 44	236 (6.3)	1,632 (4.9)	270 (5.9)	2,279 (5.0)
15 – 29	118 (3.2)	669 (2.0)	105 (2.3)	809 (1.8)
≤15	66 (1.8)	424 (1.3)	54 (1.2)	420 (0.9)
**Current medication**				
ACE-inhibitor or ARB	937 (25.1)	8,085 (24.4)	807 (17.7)	9,866 (21.7)
Aspirin*	-	-	456 (10.0)	5,036 (11.1)
Other antiplatelet agent*	-	-	375 (8.2)	2,960 (6.5)
Beta-blockers*	-	-	797 (17.5)	8,517 (18.7)
Immunosuppressants	93 (2.5)	767 (2.3)	24 (0.5)	397 (0.9)
Loop diuretic*	-	-	567 (12.4)	4,865 (10.7)
Mineralocorticoid receptor antagonist*	-	-	130 (2.9)	1,202 (2.6)
Novel oral anticoagulant*	-	-	296 (6.5)	3,154 (6.9)
Warfarin*	-	-	88 (1.9)	1,150 (2.5)

Values are mean ± SD, n (%), or median (interquartile range). Abbreviations: ACE-inhibitor – angiotensin converting enzyme inhibitor; ARB – angiotensin receptor blocker; CKD – chronic kidney disease; eGFR – estimated glomerular filtration rate (ml/min/1.73 m^2); SIMD – Scottish index of multiple deprivation. *data not available for Cohort 1.

Supplementary Table 3. Clinical characteristics of patients included in Cohorts 1 and 2, grouped by CKD status.

	COHORT 1		COHORT 2	
	**CKD**	**No CKD**	**CKD**	**No CKD**
**Number of patients, n**	**5,853**	**31,051**	**8,201**	**41,859**
**Age, years**	79 (12)	58 (20)	82 (10)	59 (19)
**Sex**				
Women	3,148 (53.8)	18,310 (59.0)	4,879 (59.5)	26,149 (62.5)
Men	2,705 (46.2)	12,741 (41.0)	3,322 (40.5)	15,710 (37.5)
**SIMD quintile**				
1 (most deprived)	719 (12.3)	4,739 (15.3)	1,322 (16.1)	8,353 (20.0)
2	1,487 (25.4)	7,667 (24.7)	1,576 (19.2)	8,011 (19.1)
3	1,033 (17.6)	5,477 (17.6)	1,673 (20.4)	7,852 (18.8)
4	1,001 (17.1)	5,392 (17.4)	2,193 (26.7)	10,837 (25.9)
5 (least deprived)	1,602 (27.4)	7,558 (24.3)	1,437 (17.5)	6,805 (16.3)
**Co-existing medical conditions**				
Angina	662 (11.3)	1,255 (4.0)	602 (7.3)	1,058 (2.5)
Atrial fibrillation*	-	-	1,651 (20.1)	2,218 (5.3)
Myocardial infarction	931 (15.9)	1,943 (6.3)	835 (10.2)	1,335 (3.2)
Heart failure	1,348 (23.0)	1,403 (4.5)	980 (11.9)	824 (2.0)
Stroke	831 (14.2)	1,986 (6.4)	794 (9.7)	1,691 (4.0)
Diabetes	2,094 (35.8)	4,083 (13.1)	2,665 (32.5)	6,420 (15.3)
Cancer	1,531 (26.2)	5,757 (18.5)	956 (11.7)	2,569 (6.1)
Chronic lower respiratory disease	1,641 (28.0)	8,220 (26.5)	2,109 (25.7)	8,431 (20.1)
Chronic liver disease	240 (4.1)	1,061 (3.4)	192 (2.3)	630 (1.5)
**Renal history**				
End-stage kidney disease	337 (5.8)	-	240 (2.9)	-
Baseline eGFR	42 (14)	91 (18)	44 (12)	94 (19)
Baseline eGFR category				
≥90	-	15,486 (49.9)	-	22,784 (54.4)
60 – 89	-	15,565 (50.1)	-	19,075 (45.6)
45 – 59	2,708 (46.3)	-	4,264 (52.0)	-
30 – 44	1,868 (31.9)	-	2,549 (31.1)	-
15 – 29	787 (13.4)	-	914 (11.1)	-
≤15	490 (8.4)	-	474 (5.8)	-
**Current medication**				
ACE-inhibitor or ARB	2,389 (40.8)	6,633 (21.4)	2,913 (35.5)	7,760 (18.5)
Aspirin*	-	-	1,756 (21.4)	3,736 (8.9)
Other antiplatelet agent*	-	-	989 (12.1)	2,346 (5.6)
Beta-blockers*	-	-	2,955 (36.0)	6,359 (15.2)
Immunosuppressants	228 (3.9)	632 (2.0)	162 (2.0)	259 (0.6)
Loop diuretic*	-	-	2,615 (31.9)	2,817 (6.7)
Mineralocorticoid receptor antagonist*	-	-	624 (7.6)	708 (1.7)
Novel oral anticoagulant*	-	-	1,278 (15.6)	2,172 (5.2)
Warfarin*	-	-	542 (6.6)	696 (1.7)

Values are mean ± SD, n (%), or median (interquartile range). Abbreviations: ACE-inhibitor – angiotensin converting enzyme inhibitor; ARB – angiotensin receptor blocker; CKD – chronic kidney disease; eGFR – estimated glomerular filtration rate (ml/min/1.73 m^2); SIMD – Scottish index of multiple deprivation. *data not available for Cohort 1.

Supplementary Table 4. Clinical characteristics of patients included in Cohorts 1 and 2, grouped according to COVID-19 and CKD status.

	**COHORT 1**				**COHORT 2**			
	**COVID-19 positive**		**COVID-19 negative**		**COVID-19 positive**		**COVID-19 negative**	
	**CKD**	**No CKD**	**CKD**	**No CKD**	**CKD**	**No CKD**	**CKD**	**No CKD**
**Number of patients, n**	**734**	**2,997**	**5,119**	**28,054**	**865**	**3,691**	**7,336**	**38,168**
**Age, years**	81 (11)	59 (21)	79 (12)	57 (20)	84 (10)	59 (20)	82 (10)	59 (19)
**Sex**								
Women	384 (52.3)	1,726 (57.6)	2,764 (54.0)	16,584 (59.1)	502 (58.0)	2,453 (66.5)	4,377 (59.7)	23,696 (62.1)
Men	350 (47.7)	1,271 (42.4)	2,355 (46.0)	11,470 (40.9)	363 (42.0)	1,238 (33.5)	2,959 (40.3)	14,472 (37.9)
**SIMD quintile**								
1 (most deprived)	103 (14.0)	476 (15.9)	616 (12.0)	4,263 (15.2)	162 (18.7)	831 (22.5)	1,160 (15.8)	7,522 (19.7)
2	207 (28.2)	795 (26.5)	1,280 (25.0)	6,872 (24.5)	185 (21.4)	747 (20.2)	1,391 (19.0)	7,264 (19.0)
3	113 (15.4)	532 (17.8)	920 (18.0)	4,945 (17.6)	182 (21.0)	665 (18.0)	1,491 (20.3)	7,187 (18.8)
4	112 (15.3)	519 (17.3)	889 (17.4)	4,873 (17.4)	216 (25.0)	905 (24.5)	1,977 (26.9)	9,932 (26.0)
5 (least deprived)	198 (27.0)	675 (22.0)	1,404 (27.4)	6,883 (24.5)	120 (13.9)	543 (14.7)	1,317 (18.0)	6,262 (16.4)
**Co-existing medical conditions**								
Angina	91 (12.4)	125 (4.2)	571 (11.2)	1,130 (4.0)	69 (8.0)	98 (2.7)	533 (7.3)	960 (2.5)
Atrial fibrillation*	-	-	-	-	207 (23.9)	215 (5.8)	1,444 (19.7)	2,003 (5.2)
Myocardial infarction	124 (16.9)	178 (5.9)	807 (15.8)	1,765 (6.3)	91 (10.5)	96 (2.6)	744 (10.1)	1,239 (3.2)
Heart failure	196 (26.7)	135 (4.5)	1,152 (22.5)	1,268 (4.5)	133 (15.4)	84 (2.3)	847 (11.5)	740 (1.9)
Stroke	127 (17.3)	257 (8.6)	704 (13.8)	1,729 (6.2)	123 (14.2)	237 (6.4)	671 (9.1)	1,454 (3.8)
Diabetes	275 (37.5)	491 (16.4)	1,819 (35.5)	3,592 (12.8)	277 (32.0)	589 (16.0)	2,388 (32.6)	5,831 (15.3)
Cancer	159 (21.7)	384 (12.8)	1,372 (26.8)	5,373 (19.2)	98 (11.3)	154 (4.2)	858 (11.7)	2,415 (6.3)
Chronic lower respiratory disease	223 (30.4)	809 (27.0)	1,418 (27.7)	7,411 (26.4)	232 (26.8)	634 (17.2)	1,877 (25.6)	7,797 (20.4)
Chronic liver disease	27 (3.7)	77 (2.6)	213 (4.2)	984 (3.5)	21 (2.4)	40 (1.1)	171 (2.3)	590 (1.5)
**Renal history**								
End-stage kidney disease	35 (4.8)	-	302 (5.9)	-	29 (3.4)	-	211 (2.9)	-
Baseline eGFR	40 (14)	90 (19)	42 (14)	91 (18)	43 (12)	93 (18)	44 (12)	94 (19)
Baseline eGFR category								
≥90	-	1,396 (46.6)	-	14,090 (50.2)	-	2,020 (54.7)	-	20,764 (54.4)
60 – 89	-	1,601 (53.4)	-	13,964 (49.8)	-	1,671 (45.3)	-	17,404 (45.6)
45 – 59	314 (42.8)	-	2,394 (46.8)	-	436 (50.4)	-	3,828 (52.2)	-
30 – 44	236 (32.2)	-	1,632 (31.9)	-	270 (31.2)	-	2,279 (31.1)	-
15 – 29	118 (16.1)	-	669 (13.1)	-	105 (12.1)	-	809 (11.0)	-
≤15	66 (9.0)	-	424 (8.3)	-	54 (6.2)	-	420 (5.7)	-
**Current medication**								
ACE-inhibitor or ARB	263 (35.8)	674 (22.5)	2,126 (41.5)	5,959 (21.2)	218 (25.2)	589 (16.0)	2,695 (36.7)	7,171 (18.8)
Aspirin*	-	-	-	-	157 (18.1)	299 (8.1)	1,599 (21.8)	3,437 (9.0)
Other antiplatelet agent*	-	-	-	-	128 (14.8)	247 (6.7)	861 (11.7)	2,099 (5.5)
Beta-blockers*	-	-	-	-	300 (34.7)	497 (13.5)	2,655 (36.2)	5,862 (15.4)

Immunosuppressants	27 (3.7)	66 (2.2)	201 (3.9)	566 (2.0)	11 (1.3)	13 (0.4)	151 (2.1)	246 (0.6)
Loop diuretic*	-	-	-	-	303 (35.0)	264 (7.1)	2,312 (31.5)	2,553 (6.7)
Mineralocorticoid receptor antagonist*	-	-	-	-	66 (7.6)	64 (1.7)	558 (7.6)	644 (1.7)
Novel oral anticoagulant*	-	-	-	-	142 (16.4)	154 (4.2)	1,136 (15.5)	2,018 (5.3)
Warfarin*	-	-	-	-	43 (5.0)	45 (1.2)	499 (6.8)	651 (1.7)

Values are mean ± SD, n (%), or median (interquartile range). Abbreviations: ACE-inhibitor – angiotensin converting enzyme inhibitor; ARB – angiotensin receptor blocker; CKD – chronic kidney disease; eGFR – estimated glomerular filtration rate (ml/min/1.73 m^2); SIMD – Scottish index of multiple deprivation.
*data not available for Cohort 1.

Supplementary Table 5. Summary of unadjusted hazard ratios and 95% confidence intervals relating to cardiovascular, all-cause, and COVID-19-related death for primary (**A**) and secondary (**B**) analyses.

			COHORT 1			COHORT 2		
			**Unadjusted Hazard Ratio**	**Lower 95% CI**	**Upper 95% CI**	**Unadjusted Hazard Ratio**	**Lower 95% CI**	**Upper 95% CI**
**A) COVID-19 positive *versus* negative**	**CKD**							
		Cardiovascular death*						
		30 days	2.65	1.97	3.58	2.42	1.83	3.20
		90 days	2.15	1.66	2.78	1.69	1.31	2.16
		End of study follow-up	1.99	1.58	2.51	1.50	1.21	1.87
		All-cause death						
		30 days	4.81	4.13	5.61	4.71	4.10	5.40
		90 days	3.33	2.92	3.80	3.14	2.78	3.54
		End of study follow-up	2.79	2.48	3.15	2.51	2.25	2.80
	**No CKD**							
		Cardiovascular death*						
		30 days	2.20	1.65	2.94	1.86	1.40	2.48
		90 days	1.81	1.40	2.34	1.48	1.16	1.91
		End of study follow-up	1.76	1.40	2.21	1.24	1.00	1.53
		All-cause death						
		30 days	4.05	3.60	4.55	4.26	3.80	4.78
		90 days	2.81	2.54	3.11	2.87	2.59	3.18
		End of study follow-up	2.25	2.05	2.46	2.03	1.85	2.22
**B) CKD *versus* no CKD**	**COVID-19 positive**							
		Cardiovascular death*						
		30 days	4.53	3.14	6.54	5.62	3.91	8.08
		90 days	4.97	3.56	6.92	5.29	3.81	7.36
		End of study follow-up	4.80	3.56	6.47	5.60	4.19	7.47
		All-cause death						
		30 days	3.03	2.59	3.54	3.70	3.19	4.30
		90 days	3.06	2.65	3.53	3.73	3.24	4.29
		End of study follow-up	3.09	2.70	3.53	3.95	3.47	4.50
		COVID-19-related death*						
		30 days	3.07	2.62	3.61	3.67	3.16	4.27
		90 days	3.10	2.65	3.61	3.69	3.19	4.27
		End of study follow-up	3.11	2.67	3.63	3.75	3.24	4.33
	**COVID-19 negative**							
		Cardiovascular death*						
		30 days	3.81	3.14	4.62	4.30	3.64	5.07

	90 days	4.36	3.74	5.08	4.83	4.26	5.48
	End of study follow-up	4.39	3.87	4.99	4.95	4.49	5.47
	All-cause death						
	30 days	2.54	2.27	2.85	3.36	3.04	3.71
	90 days	2.67	2.46	2.91	3.58	3.32	3.85
	End of study follow-up	2.58	2.41	2.76	3.57	3.37	3.78

Abbreviations: CKD – chronic kidney disease; CI – confidence interval. *Hazard ratios for cardiovascular and COVID-19-related death are cause-specific.

Supplementary Table 6. Outcomes of patients included in sensitivity analysis, in which study cohorts were restricted to patients not hospitalised either in the week before or in the two weeks following their index COVID-19 test. Patients are grouped according to COVID-19 and CKD status.

	COHORT 1				COHORT 2			
	COVID-19 positive		COVID-19 negative		COVID-19 positive		COVID-19 negative	
	CKD	No CKD	CKD	No CKD	CKD	No CKD	CKD	No CKD
**Number of patients, n**	**314**	**574**	**1,786**	**9,575**	**460**	**1,877**	**2,717**	**18,526**
**Primary outcomes**								
Cardiovascular death								
30 days	18 (5.7)	6 (1.0)	22 (1.2)	12 (0.1)	21 (4.6)	35 (1.9)	20 (0.7)	46 (0.2)
90 days	23 (7.3)	17 (3.0)	28 (1.6)	27 (0.3)	25 (5.4)	67 (3.6)	27 (1.0)	90 (0.5)
End of study follow-up	29 (9.2)	41 (7.1)	37 (2.1)	57 (0.6)	37 (8.0)	131 (7.0)	44 (1.6)	181 (1.0)
All-cause death								
30 days	99 (31.5)	29 (5.1)	144 (8.1)	112 (1.2)	141 (30.6)	95 (5.1)	193 (7.1)	156 (0.8)
90 days	120 (38.2)	61 (10.6)	182 (10.2)	208 (2.2)	161 (35.0)	200 (10.6)	220 (8.1)	306 (1.6)
End of study follow-up	138 (43.9)	125 (21.8)	215 (12.0)	371 (3.9)	192 (41.7)	401 (21.4)	274 (10.1)	673 (3.6)

Supplementary Table 7. Summary of adjusted hazard ratios and 95% confidence intervals from sensitivity analysis, in which study cohorts were restricted to patients not hospitalised either in the week before or in the two weeks following their index COVID-19 test.

			COHORT 1			COHORT 2		
			**Adjusted Hazard Ratio**	**Lower 95% CI**	**Upper 95% CI**	**Adjusted Hazard Ratio**	**Lower 95% CI**	**Upper 95% CI**
**COVID-19 positive *versus* negative**	**CKD**							
		Cardiovascular death*						
		30 days	6.70	2.19	20.51	2.30	1.10	4.81
		90 days	2.62	1.26	5.45	1.46	0.81	2.62
		End of study follow-up	2.02	1.17	3.47	1.37	0.87	2.16
		All-cause death						
		30 days	7.48	4.69	11.93	5.93	3.97	8.84
		90 days	4.04	2.87	5.69	3.37	2.50	4.53
		End of study follow-up	2.63	2.03	3.41	2.36	1.87	2.98
	**No CKD**							
		Cardiovascular death*						
		30 days	3.43	1.55	7.58	2.30	1.06	5.00
		90 days	2.35	1.32	4.16	1.50	0.84	2.69
		End of study follow-up	1.66	1.09	2.54	1.35	0.87	2.09
		All-cause death						
		30 days	3.93	2.94	5.25	6.69	4.58	9.77
		90 days	2.68	2.14	3.35	3.91	2.95	5.18
		End of study follow-up	1.82	1.52	2.19	2.41	1.96	2.97

Abbreviations: CKD – chronic kidney disease; CI – confidence interval. *Hazard ratios for cardiovascular death are cause-specific.

Supplementary Table 8. Outcomes of patients included in Cohorts 1 and 2, grouped according to COVID-19 and CKD status.

	COHORT 1				COHORT 2			
	COVID-19 positive		COVID-19 negative		COVID-19 positive		COVID-19 negative	
	CKD	No CKD	CKD	No CKD	CKD	No CKD	CKD	No CKD
**Number of patients, n**	**734**	**2,997**	**5,119**	**28,054**	**865**	**3,691**	**7,336**	**38,168**
**Primary outcomes**								
Cardiovascular death								
30 days	57 (7.8)	57 (1.9)	172 (3.4)	254 (0.9)	62 (7.2)	55 (1.5)	254 (3.5)	316 (0.8)
90 days	72 (9.8)	68 (2.3)	290 (5.7)	377 (1.3)	72 (8.3)	70 (1.9)	453 (6.2)	515 (1.3)
End of follow-up	86 (11.7)	86 (2.9)	426 (8.3)	542 (1.9)	92 (10.6)	94 (2.5)	730 (10.0)	840 (2.2)
COVID-19-related death								
30 days	250 (34.1)	377 (12.6)	-	-	295 (34.1)	397 (10.8)	-	-
90 days	267 (36.4)	404 (13.5)	-	-	313 (36.2)	423 (11.5)	-	-
End of follow-up	270 (36.8)	407 (13.6)	-	-	318 (36.8)	427 (11.6)	-	-
All-cause death								
30 days	260 (35.4)	399 (13.3)	442 (8.6)	974 (3.5)	302 (34.9)	405 (11.0)	642 (8.8)	1,025 (2.7)
90 days	304 (41.4)	473 (15.8)	803 (15.7)	1,702 (6.1)	342 (39.5)	466 (12.6)	1,157 (15.8)	1,782 (4.7)
End of follow-up	346 (47.1)	541 (18.1)	1,246 (24.3)	2,688 (9.6)	397 (45.9)	547 (14.8)	1,880 (25.6)	3,007 (7.9)
**Secondary outcomes**								
Fatal/non-fatal myocardial infarction								
30 days	7 (1.0)	8 (0.3)	132 (2.6)	254 (0.9)	9 (1.0)	NA	154 (2.1)	386 (1.0)
90 days	8 (1.1)	9 (0.3)	155 (3.0)	293 (1.0)	9 (1.0)	9 (0.2)	193 (2.6)	428 (1.1)
End of follow-up	11 (1.5)	15 (0.5)	190 (3.7)	351 (1.3)	13 (1.5)	11 (0.3)	248 (3.4)	505 (1.3)
Fatal myocardial infarction								
End of follow-up	NA	NA	62 (1.2)	74 (0.3)	7 (0.8)	10 (0.3)	113 (1.5)	142 (0.4)
Fatal/non-fatal heart failure								
30 days	16 (2.2)	NA	117 (2.3)	133 (0.5)	21 (2.4)	6 (0.2)	348 (4.7)	362 (0.9)
90 days	21 (2.9)	8 (0.3)	168 (3.3)	166 (0.6)	23 (2.7)	8 (0.2)	423 (5.8)	414 (1.1)
End of follow-up	24 (3.3)	10 (0.3)	226 (4.4)	212 (0.8)	28 (3.2)	14 (0.4)	496 (6.8)	478 (1.3)
Fatal heart failure								
End of follow-up	18 (2.5)	NA	88 (1.7)	60 (0.2)	20 (2.3)	11 (0.3)	182 (2.5)	93 (0.2)
Fatal/non-fatal stroke								

30 days	10 (1.4)	18 (0.6)	96 (1.9)	348 (1.2)	15 (1.7)	31 (0.8)	249 (3.4)	574 (1.5)
90 days	13 (1.8)	23 (0.8)	121 (2.4)	415 (1.5)	19 (2.2)	38 (1.0)	294 (4.0)	648 (1.7)
End of follow-up	16 (2.2)	28 (0.9)	158 (3.1)	487 (1.7)	25 (2.9)	44 (1.2)	348 (4.7)	744 (1.9)
Fatal stroke								
End of follow-up	6 (0.8)	14 (0.5)	57 (1.1)	135 (0.5)	NA	NA	263 (3.6)	410 (1.1)
Fatal/non-fatal pulmonary embolism								
End of follow-up	NA	15 (0.5)	60 (1.2)	286 (1.0)	7 (0.8)	17 (0.5)	87 (1.2)	370 (1.0)
Fatal pulmonary embolism								
End of follow-up	NA	NA	11 (0.2)	20 (0.1)	NA	NA	11 (0.1)	41 (0.1)
Atrial fibrillation hospitalisations								
30 days	NA	7 (0.2)	189 (3.7)	461 (1.6)	7 (0.8)	10 (0.3)	259 (3.5)	626 (1.6)
90 days	12 (1.6)	13 (0.4)	225 (4.4)	557 (2.0)	12 (1.4)	13 (0.3)	303 (4.1)	714 (1.9)
End of follow-up	15 (2.0)	18 (0.6)	292 (5.7)	676 (2.4)	24 (2.8)	24 (0.6)	435 (5.9)	939 (2.5)
Cardiovascular hospitalisations								
30 days	28 (3.8)	53 (1.8)	630 (12.3)	1,847 (6.6)	41 (4.7)	80 (2.2)	1,311 (17.9)	3,076 (8.1)
90 days	41 (5.6)	82 (2.7)	765 (14.9)	2,175 (7.8)	49 (5.7)	100 (2.7)	1,483 (20.2)	3,381 (8.9)
End of follow-up	54 (7.4)	105 (3.5)	958 (18.7)	2,549 (9.1)	60 (6.9)	119 (3.2)	1,636 (22.3)	3,685 (9.7)
All hospitalisations								
30 days	274 (37.3)	896 (29.9)	2,928 (57.2)	13,361 (47.6)	359 (41.5)	899 (24.4)	5,063 (69.0)	18,562 (48.6)
90 days	335 (45.6)	1,128 (37.6)	3,408 (66.6)	15,767 (56.2)	388 (44.9)	970 (26.3)	5,267 (71.8)	19,242 (50.4)
End of follow-up	386 (52.6)	1,311 (43.7)	3,887 (75.9)	17,952 (64.0)	411 (47.5)	1,043 (28.3)	5,429 (74.0)	19,911 (52.2)
Length of stay, median [IQR]	12 [5, 24]	7 [3, 18]	5 [2, 12]	3 [1, 7]	9 [4, 17]	5 [2, 15]	6 [2, 16]	2 [0, 7]

Values are mean ± SD, n (%), or median (interquartile range). NAs represent redacted count data <5.

Supplementary Figures

Supplementary Figure 1. Flow diagrams for Cohorts 1 (**a**) and 2 (**b**). Abbreviations: AKI – acute kidney injury; CKD – chronic kidney disease; eGFR – estimated glomerular filtration rate (ml/min/1.73 m^2); KTx – kidney transplant; HD – haemodialysis; PD – peritoneal dialysis; RRT – renal replacement therapy.

a) Cohort 1

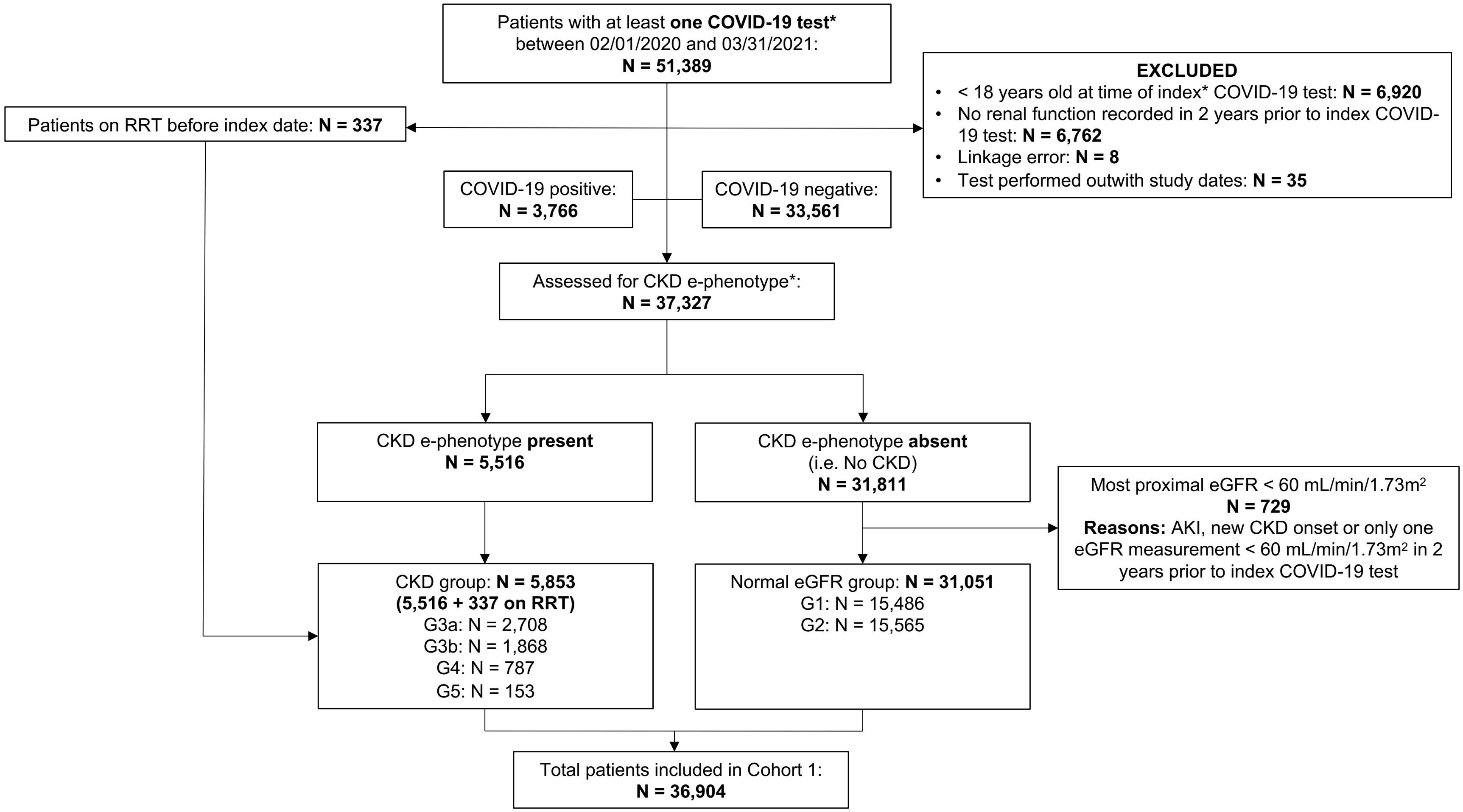

*See **Methods** for definitions. Abbreviations: AKI – acute kidney injury CKD – chronic kidney disease; eGFR – estimated glomerular filtration rate; KTx – kidney transplant; HD – hemodialysis; PD – peritoneal dialysis; RRT – renal replacement therapy;

b) Cohort 2

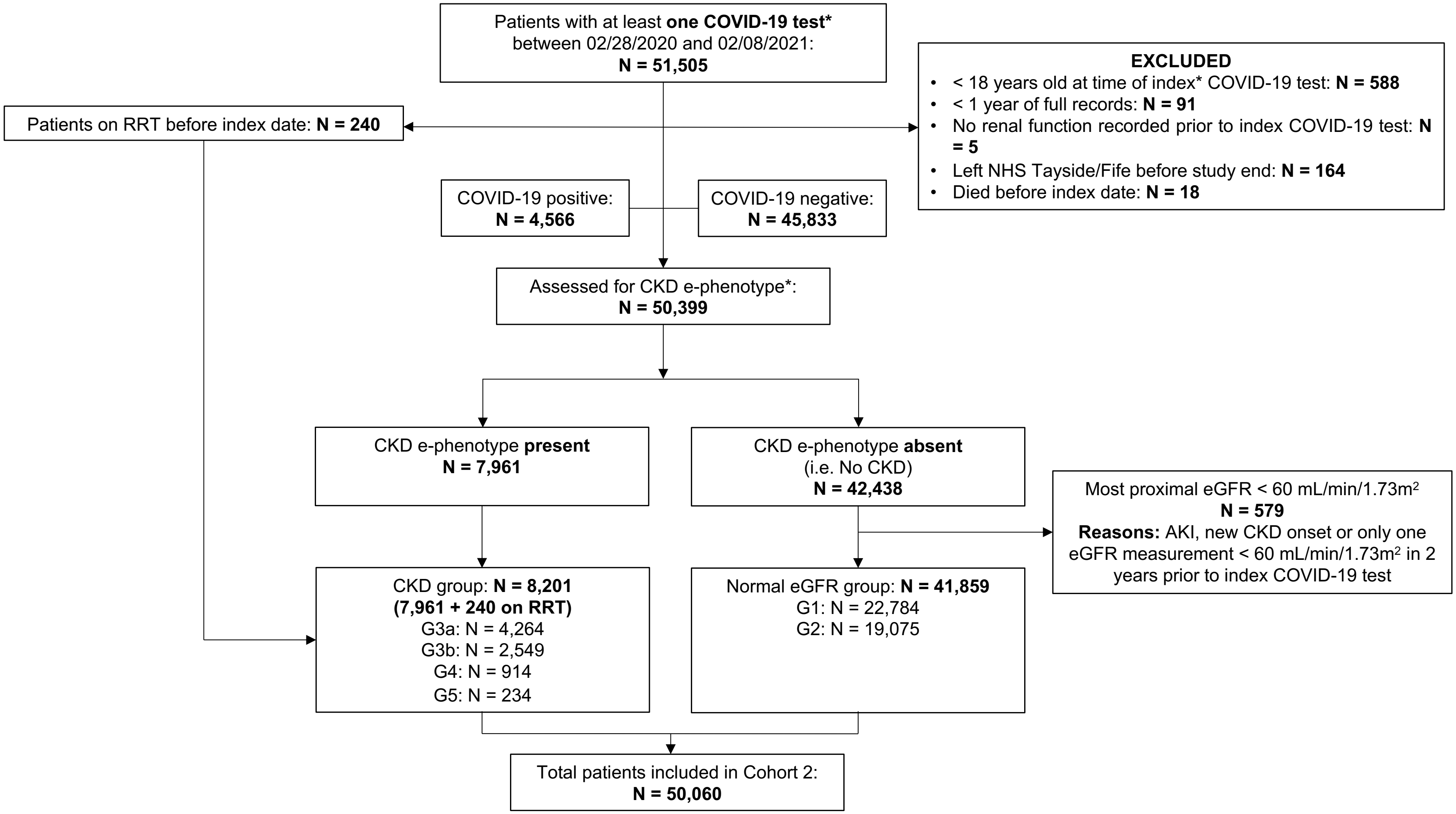

*See **Methods** for definitions. Abbreviations: AKI – acute kidney injury CKD – chronic kidney disease; eGFR – estimated glomerular filtration rate; KTx – kidney transplant; HD – hemodialysis; PD – peritoneal dialysis; RRT – renal replacement therapy;

Supplementary Figure 2. Love plots summarising the effect of covariate balancing according to COVID-19 status (i.e. positive *versus* negative) (**a**) in patients with CKD (**Panel A**: Cohort 1; **Panel C**: Cohort 2) and in patients without CKD (**Panel B**: Cohort 1; **Panel D**: Cohort 2), and the effect of covariate balancing according to CKD status (i.e. CKD *versus* no CKD) (**b**) in patients with COVID-19 (**Panel A**: Cohort 1; **Panel C**: Cohort 2) and in patients without COVID-19 (**Panel B**: Cohort 1; **Panel D**: Cohort 2). Abbreviations: ACE-inhibitor – angiotensin converting enzyme inhibitor; ARB – angiotensin receptor blocker; SIMD – Scottish index of multiple deprivation.

a) Covariate balancing according to COVID-19 status

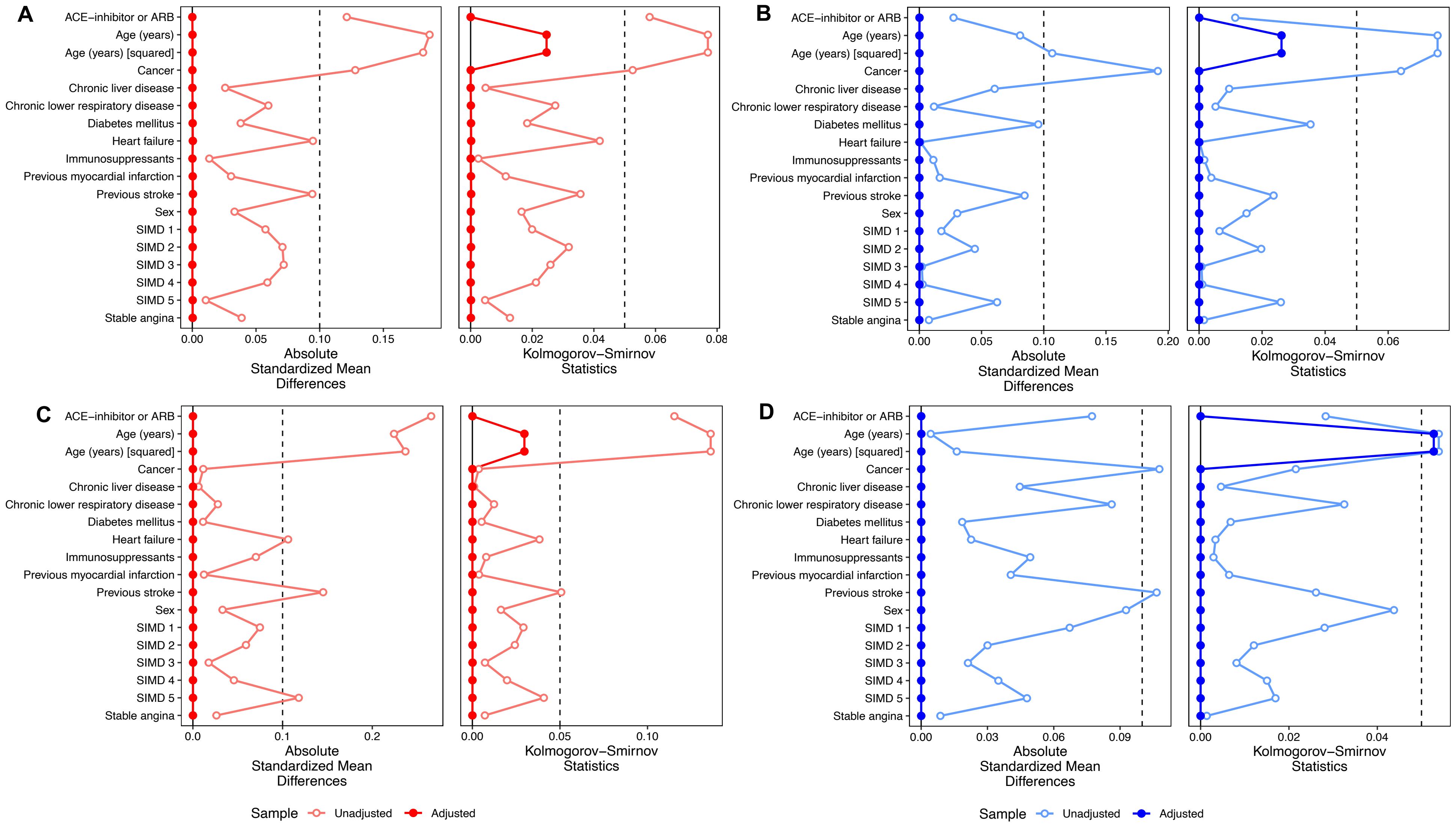

b) Covariate balancing according to CKD status

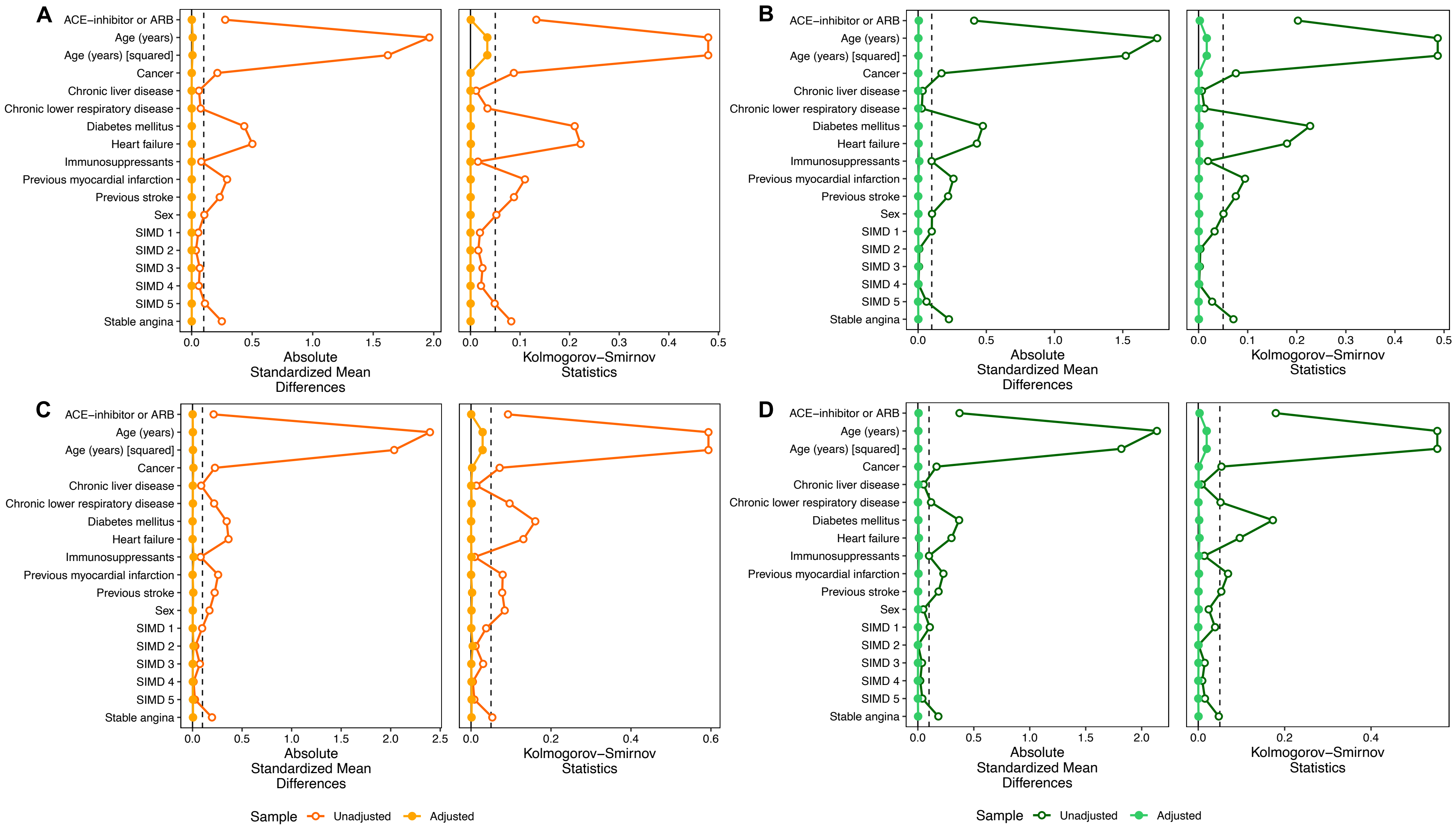

Supplementary Figure 3. Survival curves for cardiovascular (**a**) and all-cause death (**b**) according to CKD status (i.e. CKD *versus* no CKD) for patients with COVID-19 (**Panel A**: Cohort 1; **Panel C**: Cohort 2) and patients without COVID-19 (**Panel B**: Cohort 1; **Panel D**: Cohort 2).

a) Cardiovascular death

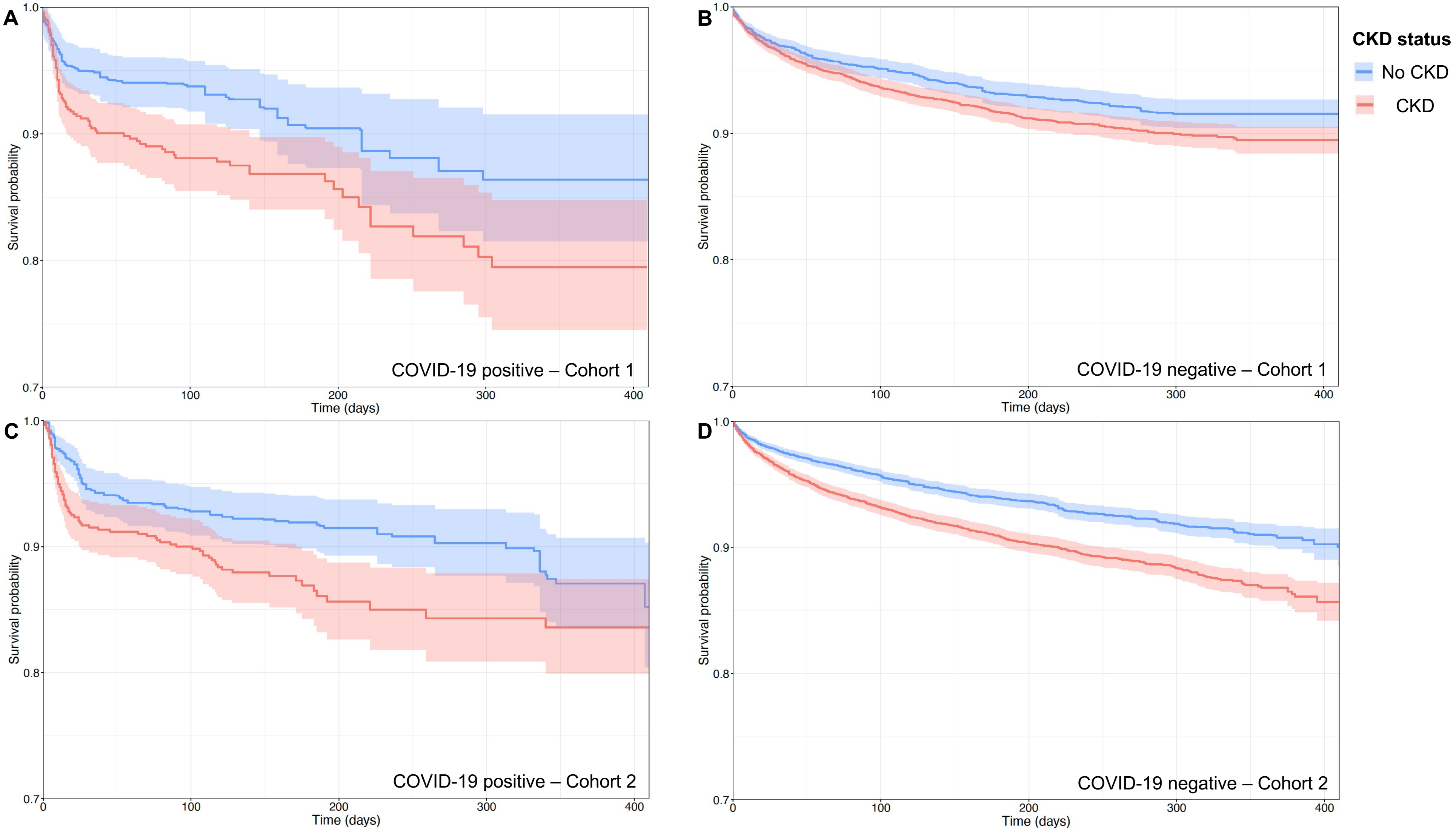

b) All-cause death

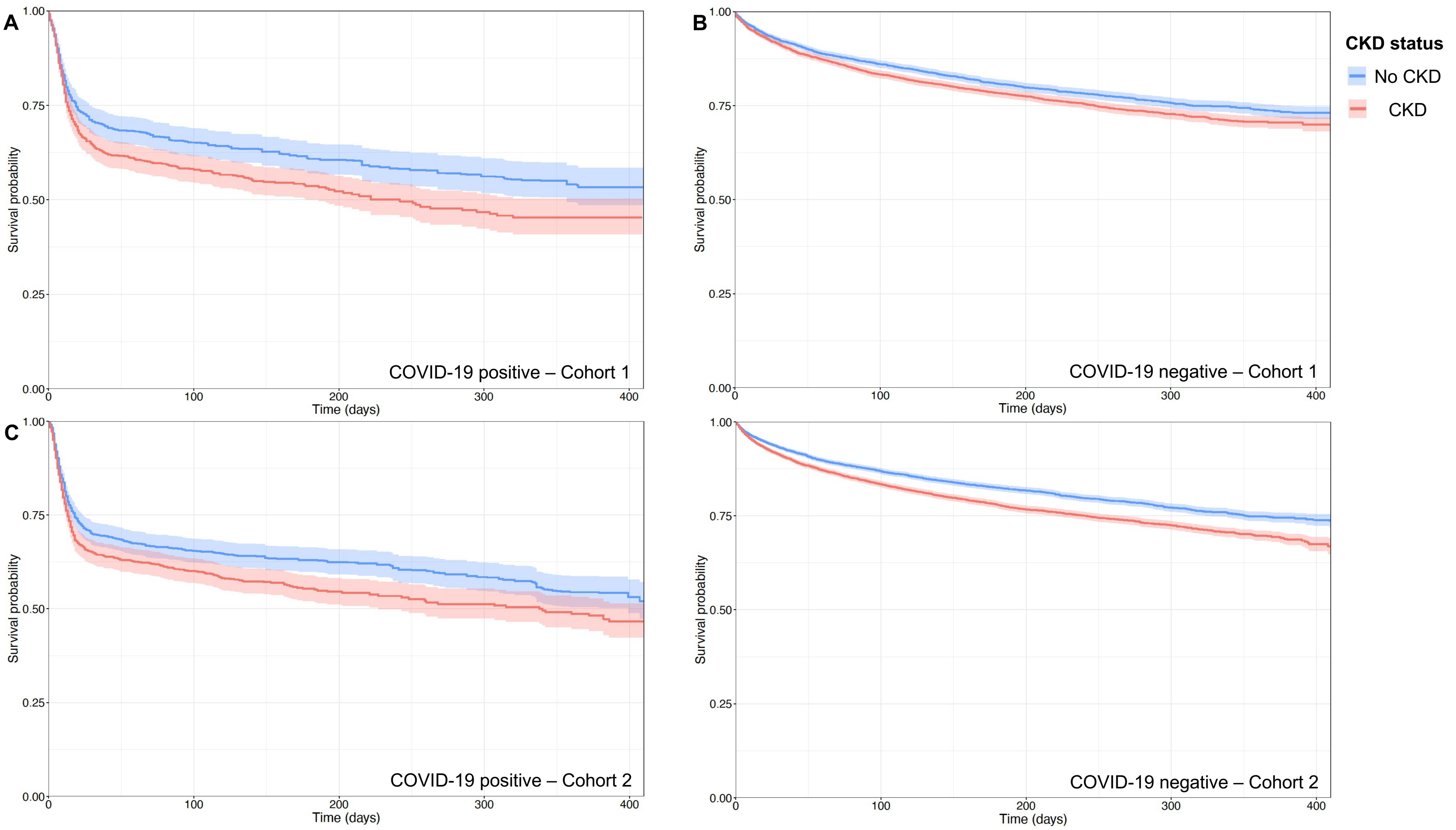

Supplementary Figure 4. Forest plot summarising adjusted hazard ratios (HR) from Cohorts 1 and 2 and associated pooled meta-estimates for cardiovascular (**a**) and all-cause death (**b**) according to CKD status (i.e. CKD *versus* No CKD) for patients with COVID-19 (**red**) and for patients without COVID-19 (**blue**) at 30 days (**top panel**), 90 days (**middle panel**) and to the end of study follow-up (**bottom panel**).

a) Cardiovascular death

	HR	lower	upper
COVID positive – 30 days			
Cohort 1	1.71	1.04	2.82
Cohort 2	1.70	1.14	2.54
Summary	1.71	1.25	2.33
COVID negative – 30 days			
Cohort 1	1.17	0.92	1.48
Cohort 2	1.58	1.30	1.92
Summary	1.40	1.20	1.63
COVID positive – 90 days			
Cohort 1	1.90	1.21	2.97
Cohort 2	1.62	1.12	2.33
Summary	1.73	1.30	2.29
COVID negative – 90 days			
Cohort 1	1.31	1.08	1.58
Cohort 2	1.66	1.43	1.92
Summary	1.52	1.35	1.71
COVID positive – study end			
Cohort 1	1.69	1.15	2.47
Cohort 2	1.61	1.17	2.21
Summary	1.64	1.29	2.10
COVID negative – study end			
Cohort 1	1.29	1.10	1.50
Cohort 2	1.56	1.39	1.75
Summary	1.46	1.33	1.60

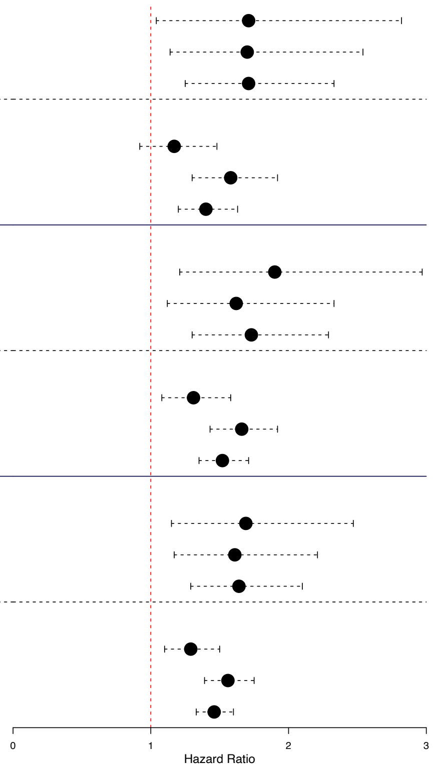

b) All-cause death

	HR	lower	upper
COVID positive – 30 days			
Cohort 1	1.25	1.04	1.52
Cohort 2	1.21	1.02	1.43
Summary	1.23	1.08	1.39
COVID negative – 30 days			
Cohort 1	1.16	1.01	1.34
Cohort 2	1.31	1.16	1.47
Summary	1.25	1.14	1.36
COVID positive – 90 days			
Cohort 1	1.27	1.06	1.51
Cohort 2	1.21	1.04	1.42
Summary	1.24	1.10	1.39
COVID negative – 90 days			
Cohort 1	1.21	1.09	1.34
Cohort 2	1.32	1.21	1.44
Summary	1.27	1.19	1.36
COVID positive – study end			
Cohort 1	1.27	1.08	1.51
Cohort 2	1.24	1.07	1.43
Summary	1.25	1.12	1.41
COVID negative – study end			
Cohort 1	1.17	1.08	1.27
Cohort 2	1.31	1.22	1.41
Summary	1.24	1.18	1.31

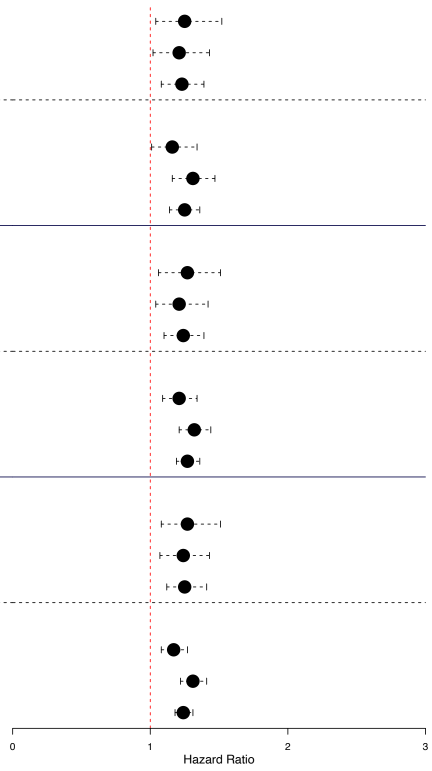

Supplementary Figure 5. Adjusted hazard ratios of cardiovascular (**a**) and all-cause death (**b**) according to eGFR (ml/min/1.73 m^2), in patients with COVID-19 (**Panel A**: Cohort 1; **Panel C**: Cohort 2) and in patients without COVID-19 (**Panel B**: Cohort 1; **Panel D**: Cohort 2).

a) Cardiovascular death

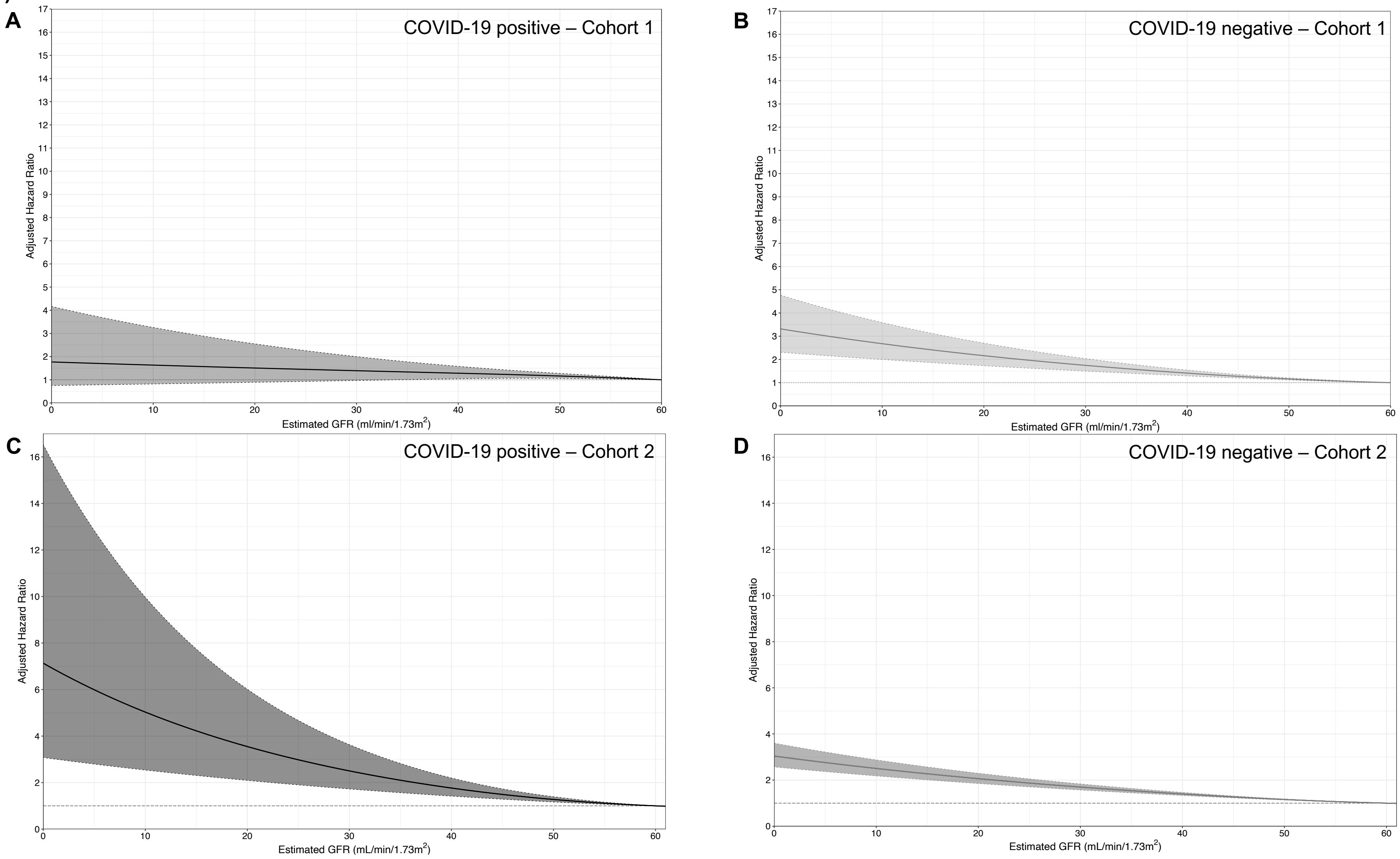

b) All-cause death

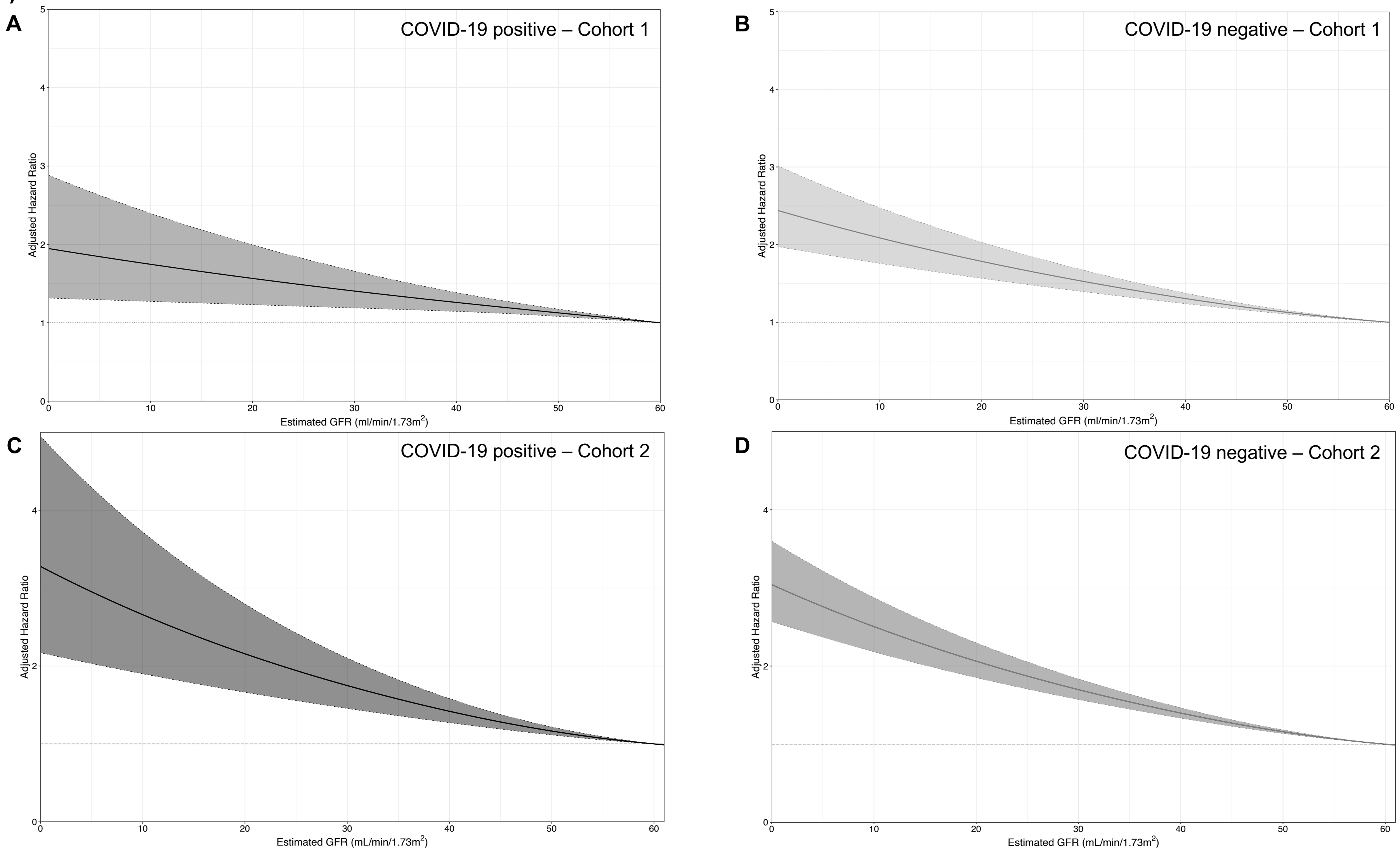

Supplementary Figure 6. Forest plot summarising adjusted hazard ratios (HR) from Cohorts 1 and 2 and associated pooled meta-estimates for COVID-19-related death according to CKD status (i.e. CKD *versus* no CKD) at 30 days (**top panel**), 90 days (**middle panel**) and to the end of study follow-up (**bottom panel**).

	HR	lower	upper
**30 days**			
Cohort 1	1.32	1.09	1.61
Cohort 2	1.20	1.01	1.42
Summary	1.25	1.10	1.42
**90 days**			
Cohort 1	1.35	1.12	1.63
Cohort 2	1.20	1.02	1.41
Summary	1.26	1.12	1.43
**Study end**			
Cohort 1	1.36	1.12	1.64
Cohort 2	1.20	1.02	1.42
Summary	1.27	1.12	1.43

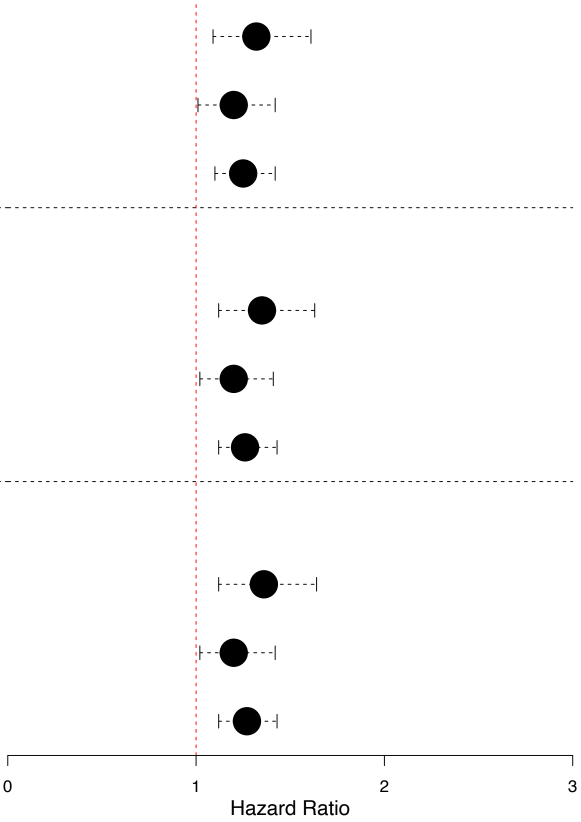

Supplementary Figure 7. Adjusted hazard ratios of COVID-19-related death according to estimated glomerular filtration rate (ml/min/1.73 m^2) in Cohort 1 (**a**) and Cohort 2 (**b**).

a) Cohort 1

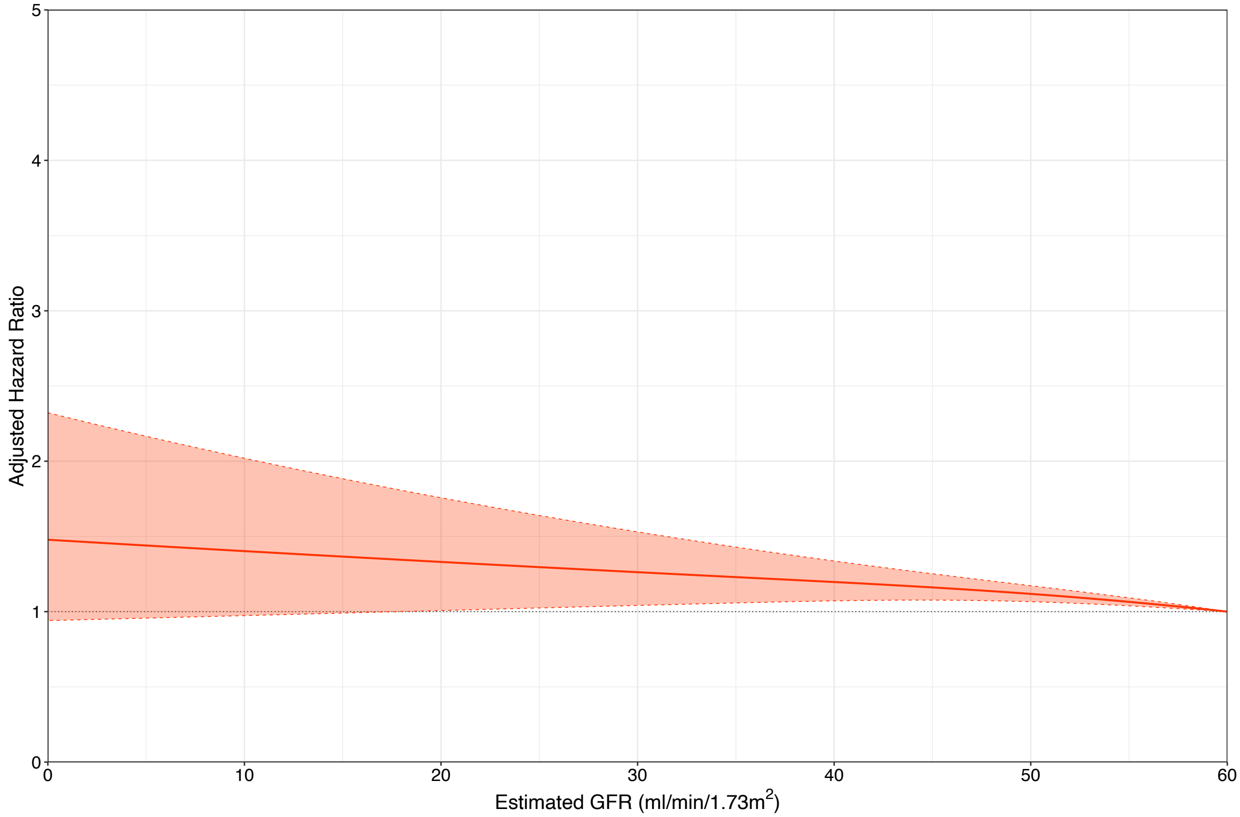

b) Cohort 2

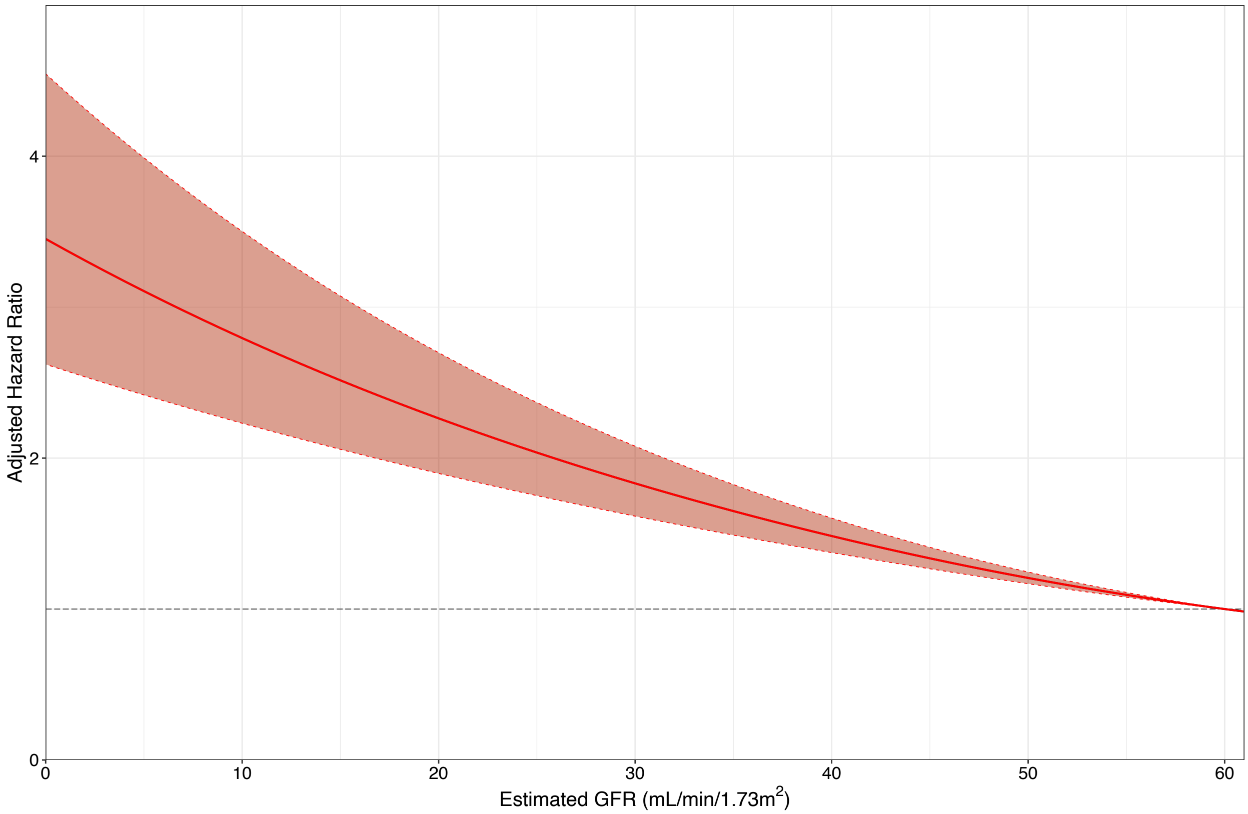

References

1. SMR Datasets - SMR Validation Section [Available from: https://www.ndc.scot.nhs.uk/Data-Dictionary/SMR-Datasets/SMR-Validation-Section/.
2. Assessment of SMR01 Data Scotland 2014-2015 [Available from: https://www.isdscotland.org/Products-and-Services/Data-Quality/docs/Assessment-of-SMR01-Data-2014-15-report-180508.pdf.
3. Shah ASV, McAllister DA, Gallacher P, Astengo F, Rodriguez Perez JA, Hall J, et al. Incidence, Microbiology, and Outcomes in Patients Hospitalized With Infective Endocarditis. Circulation. 2020;141(25):2067-77.
4. National Records of Scotland - Vital Events - Deaths [Available from: https://www.nrscotland.gov.uk/files/statistics/vital-events/ve-deaths-cause-of-death-text-codes.pdf.
5. Wild S, Fischbacher C, McKnight J. Using Large Diabetes Databases for Research. Journal of Diabetes Science and Technology. 2016;10(5):1073-8.
6. Government S. Scottish Index of Multiple Deprivation 2020 2020 [updated 28/01/2020. Available from: https://www.gov.scot/publications/scottish-index-multiple-deprivation-2020/pages/1/.
7. Scottish Index of Multiple Deprivation 2020 [Available from: https://www.gov.scot/collections/scottish-index-of-multiple-deprivation-2020/.
8. Norton JM, Ali K, Jurkovitz CT, Kiryluk K, Park M, Kawamoto K, et al. Development and validation of a pragmatic electronic phenotype for CKD. Clinical Journal of the American Society of Nephrology. 2019;14(9):1306-14.
9. Levey AS, Stevens LA, Schmid CH, Zhang Y, Castro III AF, Feldman HI, et al. A new equation to estimate glomerular filtration rate. Annals of internal medicine. 2009;150(9):604-12.
10. Eknoyan G, Lameire N, Eckardt K, Kasiske B, Wheeler D, Levin A, et al. KDIGO 2012 clinical practice guideline for the evaluation and management of chronic kidney disease. Kidney Int. 2013;3(1):5-14.
11. Scottish Care Information Diabetes Collaboration [Available from: https://www.sci-diabetes.scot.nhs.uk/.
12. Funk MJ, Westreich D, Wiesen C, Stürmer T, Brookhart MA, Davidian M. Doubly robust estimation of causal effects. American journal of epidemiology. 2011;173(7):761-7.
13. Li X, Shen C. Doubly robust estimation of causal effect: upping the odds of getting the right answers. Circulation: Cardiovascular Quality and Outcomes. 2020;13(1):e006065.
14. Imai K, Ratkovic M. Covariate balancing propensity score. Journal of the Royal Statistical Society: Series B (Statistical Methodology). 2014;76(1):243-63.
15. Williams B, Zhang Y. Hypertension, renin–angiotensin–aldosterone system inhibition, and COVID-19. The Lancet. 2020;395(10238):1671-3.
16. Thng ZX, De Smet MD, Lee CS, Gupta V, Smith JR, McCluskey PJ, et al. COVID-19 and immunosuppression: a review of current clinical experiences and implications for ophthalmology patients taking immunosuppressive drugs. British Journal of Ophthalmology. 2021;105(3):306-10.
17. Royston P, Sauerbrei W. Multivariable modeling with cubic regression splines: a principled approach. The Stata Journal. 2007;7(1):45-70.
18. The Strengthening the Reporting of Observational Studies in Epidemiology (STROBE) Statement: guidelines for reporting observational studies. Available from: https://www.equator-network.org/reporting-guidelines/strobe/ . Accessed 11/02/2021. [cited 2021. Available from: https://www.equator-network.org/reporting-guidelines/strobe/.
