# Supplementary material for: Cardiovascular outcomes in patients with chronic kidney disease and COVID-19: a multi-regional data-linkage study
Source: Eur Respir J. 2022 Nov 10;60(5):2103168. doi: 10.1183/13993003.03168-2021 (PMC9101552; doi:10.1183/13993003.03168-2021)

Cardiovascular outcomes in patients with chronic kidney disease & COVID-19: a multi-regional data-linkage study

Lambourg & Gallacher *et al*, 2021

Methods

SARS-CoV-2 PCR

86,964 patients
with prior measures of serum creatinine tested in Scotland, UK

Cohort 1: N = 36,904
CKD = 15.9%

Cohort 2: N = 50,060
CKD = 16.4%

Primary outcomes

- Cardiovascular death
- All-cause death

Covariate Balancing Propensity Score
Weighted comparison by test status

SARS-CoV-2 ⊖ ⊕ → in CKD / in non-CKD

Results

CKD

- SARS-CoV-2 ⊕ : **11.4%**
- Outcomes in patients with COVID-19:
  - Cardiovascular death: **11.1%**
  - COVID-19 death: **36.8%**
  - All-cause death: **46.5%**

No CKD

- SARS-CoV-2 ⊕: **9.2%**
- Outcomes in patients with COVID-19:
  - Cardiovascular death: **2.7%**
  - COVID-19 death: **12.5%**
  - All-cause death: **16.3%**

COVID-19 ⊕ *vs* ⊖ → EXCESS RISK in PATIENTS WITH CKD

Adjusted meta-estimates combining both cohorts for cardiovascular and all-cause death

COVID-19 vs no COVID-19

Cardiovascular death:

	Hazard ratio	Lower 95% CI	Upper 95% CI
30 days	2.34	1.83	2.99
90 days	1.71	1.39	2.11
Study end	1.57	1.31	1.89

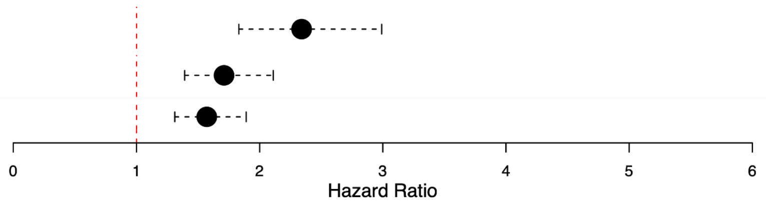

All-cause death:

	Hazard ratio	Lower 95% CI	Upper 95% CI
30 days	4.53	3.97	5.16
90 days	3.01	2.68	3.35
Study end	2.41	2.17	2.64

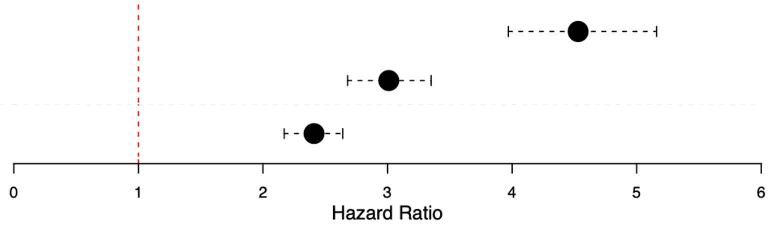

Supplement: Supplementary file 2 [file ERJ-03168-2021.Abstract.pdf]
