# Supplementary material for: Cardiovascular outcomes in patients with chronic kidney disease and COVID-19: a multi-regional data-linkage study
Source: Eur Respir J. 2022 Nov 10;60(5):2103168. doi: 10.1183/13993003.03168-2021 (PMC9101552; doi:10.1183/13993003.03168-2021)
Supplement: Supplementary file 3 [file ERJ-03168-2021.Shareable.pdf]

EUROPEAN RESPIRATORY JOURNAL
ORIGINAL RESEARCH ARTICLE
E.J. LAMBOURG ET AL.

SHAREABLE PDF

Cardiovascular outcomes in patients with chronic kidney disease and COVID-19: a multi-regional data-linkage study

Emilie J. Lambourg [1,5], Peter J. Gallacher[2,5], Robert W. Hunter[2,3], Moneeza Siddiqui [1], Eve Miller-Hodges[2,3], James D. Chalmers[4], Dan Pugh[3], Neeraj Dhaun [2,3,6] and Samira Bell [1,6]

[1]Division of Population Health and Genomics, University of Dundee, Dundee, UK. [2]BHF/University Centre for Cardiovascular Science, University of Edinburgh, Edinburgh, UK. [3]Dept of Renal Medicine, Royal Infirmary of Edinburgh, Edinburgh, UK. [4]Dept of Molecular and Clinical Medicine, University of Dundee, Dundee, UK. [5]These authors contributed equally. [6]These authors contributed equally.

Corresponding author: Neeraj Dhaun (bean.dhaun@ed.ac.uk)

Check for updates

Shareable abstract (@ERSpublications)
COVID-19 significantly increases the risk of cardiovascular complications and death in patients with CKD, especially in the short-term. COVID-19 vaccination and cardiovascular risk reduction strategies must be prioritised for all patients with CKD. https://bit.ly/3MAY7Wg

Cite this article as: Lambourg EJ, Gallacher PJ, Hunter RW, *et al*. Cardiovascular outcomes in patients with chronic kidney disease and COVID-19: a multi-regional data-linkage study. *Eur Respir J* 2022; 60: 2103168 [DOI: 10.1183/13993003.03168-2021].

This single-page version can be shared freely online.

Copyright ©The authors 2022.

This version is distributed under the terms of the Creative Commons Attribution Licence 4.0.

Received: 15 Dec 2021
Accepted: 21 April 2022

Abstract

Background Data describing cardiovascular outcomes in patients with coronavirus disease 2019 (COVID-19) and chronic kidney disease (CKD) are lacking. We compared cardiovascular outcomes of patients with and without COVID-19, stratified by CKD status.

Methods This retrospective, multi-regional data-linkage study utilised individual patient-level data from two Scottish cohorts. All patients tested for severe acute respiratory syndrome coronavirus 2 (SARS-CoV-2) in Cohort 1 between 1 February 2020 and 31 March 2021 and in Cohort 2 between 28 February 2020 and 8 February 2021 were included.

Results Overall, 86 964 patients were tested for SARS-CoV-2. There were 36 904 patients (mean±SD age 61±21 years; 58.1% women; 15.9% CKD; 10.1% COVID-19 positive) in Cohort 1 and 50 060 patients (mean±SD age 63±20 years; 62.0% women; 16.4% CKD; 9.1% COVID-19 positive) in Cohort 2. In CKD patients, COVID-19 increased the risk of cardiovascular death by more than two-fold within 30 days (cause-specific hazard ratio (csHR) meta-estimate 2.34, 95% CI 1.83–2.99) and by 57% at the end of study follow-up (csHR meta-estimate 1.57, 95% CI 1.31–1.89). Similarly, the risk of all-cause death in COVID-19 positive *versus* negative CKD patients was greatest within 30 days (HR 4.53, 95% CI 3.97–5.16). Compared with patients without CKD, those with CKD had a higher risk of testing positive (11.5% *versus* 9.3%). Following a positive test, CKD patients had higher rates of cardiovascular death (11.1% *versus* 2.7%), cardiovascular complications and cardiovascular hospitalisations (7.1% *versus* 3.3%) than those without CKD.

Conclusions COVID-19 increases the risk of cardiovascular and all-cause death in CKD patients, especially in the short-term. CKD patients with COVID-19 are also at a disproportionate risk of cardiovascular complications than those without CKD.
